# Exosomal Plasminogen Activator Inhibitor-1 Induces Ionizing Radiation-Adaptive Glioblastoma Cachexia

**DOI:** 10.3390/cells11193102

**Published:** 2022-10-01

**Authors:** Eunguk Shin, Hyunkoo Kang, Haksoo Lee, Sungmin Lee, Jaewan Jeon, Kimoon Seong, Hyesook Youn, Buhyun Youn

**Affiliations:** 1 Department of Integrated Biological Science, Pusan National University, Busan 46241, Korea; 2 Department of Radiation Oncology, Haeundae Paik Hospital, Inje University College of Medicine, Busan 48108, Korea; 3 Laboratory of Biological Dosimetry, National Radiation Emergency Medical Center (NREMC), Korea Institute of Radiological and Medical Sciences (KIRAMS), Seoul 01812, Korea; 4 Department of Integrative Bioscience and Biotechnology, Sejong University, Seoul 05006, Korea; 5 Department of Biological Sciences, Pusan National University, Busan 46241, Korea

**Keywords:** cachexia, glioblastoma, radiotherapy, PAI-1, exosome

## Abstract

Cancer cachexia is a muscle-wasting syndrome that leads to a severely compromised quality of life and increased mortality. A strong association between cachexia and poor prognosis has been demonstrated in intractable cancers, including glioblastoma (GBM). In the present study, it was demonstrated that ionizing radiation (IR), the first-line treatment for GBM, causes cancer cachexia by increasing the exosomal release of plasminogen activator inhibitor-1 (PAI-1) from glioblastoma cells. Exosomal PAI-1 delivered to the skeletal muscle is directly penetrated in the muscles and phosphorylates STAT3 to intensify muscle atrophy by activating muscle RING-finger protein-1 (MuRF1) and muscle atrophy F-box (Atrogin1); furthermore, it hampers muscle protein synthesis by inhibiting mTOR signaling. Additionally, pharmacological inhibition of PAI-1 by TM5441 inhibited muscle atrophy and rescued muscle protein synthesis, thereby providing survival benefits in a GBM orthotopic xenograft mouse model. In summary, our data delineated the role of PAI-1 in the induction of GBM cachexia associated with radiotherapy-treated GBM. Our data also indicated that targeting PAI-1 could serve as an attractive strategy for the management of GBM following radiotherapy, which would lead to a considerable improvement in the quality of life of GBM patients undergoing radiotherapy.

## 1. Introduction

In intractable cancers, 50–80% of cancer patients develop cachectic symptoms, which cause muscle and fat loss by 5% or more, loss of appetite, and metabolic imbalance due to the direct or indirect effects [1,2]. The basic treatment regimens for cachexia involve exercise therapy or dietary interventions; however, these treatment approaches are ineffectual and difficult to implement, especially in cancer patients undergoing high-intensity chemotherapy. Cancer cachexia is currently being actively studied in association with various cancers; however, an effective drug for cancer cachexia has not been developed as yet.

Glioblastoma (GBM) is a malignant WHO grade IV primary brain tumor, with an average survival period of only 15 months [3,4]. Surgical removal of the entire tumor is almost impossible, so radiotherapy and chemotherapy are essential in the treatment process [5]. According to previous reports, many GBM patients suffer from cancer cachexia, and it is known that cancer cachexia affects the survival rate of GBM patients [6,7,8,9]. In addition, the incidences of other cancer-associated cachexia have been reported to increase post radiotherapy [10,11,12,13]. Although research on treatment is actively performed [14,15,16,17], most of these remain in basic research. In improving the treatment of GBM, cachexia can also significantly affect the patient’s survival rate, and the need for additional research is also urgently suggested.

Plasminogen activator inhibitor-1 (PAI-1) is a serine protease inhibitor, and a factor involved in the regulation of intra- and extravascular fibrinolysis [18,19]. PAI-1 is upregulated in atrophic skeletal muscle [20], and this change is associated with impaired muscle regeneration [21,22]. Therefore, it can be seen that PAI-1 is closely related to inducing cancer cachexia.

Since cancer cachexia is a disease in which rapid muscle atrophy occurs due to the effects of cancer in other organs, the interaction between muscles and other organs is a very important cause of inducing cachexia. Therefore, various types of factors secreted from other organs are transported to the muscle through various pathways and could possibly induce cachexia. Exosomes are a subgroup of extracellular vesicles, which are important mediators of long-distance intercellular communication and are involved in a diverse range of biological processes [23]. The cargo proteins inside the exosome move to the target cell, causing various downstream mechanisms [24].

In existing research, no prior studies have been conducted on GBM-induced cachexia due to the poor prognosis of GBM patients. However, research on radiation-induced GBM cachexia is essential to improve the quality of life as well as the survival rate of patients after radiotherapy. To elucidate the mechanism and key molecules involved in the intercellular communication that mediates irradiation-induced GBM cachexia, secretome from IR-irradiated GBM cells were investigated in the present study. Radiation-dependent secretion of PAI-1 from irradiated GBM cells demonstrated the effects of both activating muscle atrophy (catabolic pathway) and disrupting muscle protein synthesis (anabolic pathway) in skeletal muscle cells. This research has observed that upregulated PAI-1 in the irradiated GBM-derived secretome provides the possibility of cancer cachexia development in GBM patients who are treated with radiotherapy. Furthermore, the obtained findings showed that PAI-1 could serve as a promising therapeutic target for enhancing the efficiency of cancer cachexia management in improving the treatment outcomes of GBM patients post-radiotherapy.

## 2. Materials and Methods

### 2.1. Chemicals, Antibodies, and Reagents

TM5441 was purchased from Cayman Chemical (Ann Arbor, MI, USA). STAT3 inhibitor III (AG 490) was purchased from Sigma-Aldrich (WP1066, CAS 857064-38-1, St. Louis, MO, USA). Primary antibodies specific to Vinculin were purchased from Cell Signaling Technology (Cell Signaling Technology, Beverly, MA, USA). Primary antibodies specific to CD9, CD63, MHCI, mTOR, PAI-1, MuRF1, Atrogin-1, p-AKT, AKT, p-STAT3, and STAT3 were purchased from Santa Cruz Biotechnology (Santa Cruz Biotechnology, Santa Cruz, CA, USA). Primary antibodies specific to p-mTOR (Ser2448) and NeuN were purchased from Abcam (Abcam, Cambridge, MA, USA). A primary antibody specific to PSD95 was purchased from Invitrogen (Invitrogen, Carlsbad, CA, USA). Secondary antibodies specific to mouse IgG and rabbit IgG were purchased from Enzo Life Sciences (Enzo Life Sciences, Ann Arbor, MI, USA). Eagle’s Minimum Essential Medium (MEM), Dulbecco’s Modified Eagle Medium (DMEM), Phosphate Buffered Saline (PBS), and fetal bovine serum (FBS) were acquired from WelGENE, Inc. (WelGENE Inc., Daegu, Korea). Penicillin, streptomycin, TRIzol were obtained from Thermo Fisher Scientific (Thermo Fisher Scientific, Cleveland, OH, USA). SiRNAs specific to human PAI-1 and control siRNA were purchased from Bioneer (Bioneer, Daejeon, Korea). Plasmid for PAI-1-GFP was purchased from Sino Biological (HG10296-ACG, Beijing, China).

### 2.2. Cell Lines, Cell Culture, and Irradiation

U87MG cell line was obtained from the Korea Cell Line Bank (KCLB, Seoul, Republic of Korea). The phenotypes of these cell lines have been authenticated by the KCLB. The cells were grown in a MEM medium consisting of 10% FBS, 100 U/mL penicillin, and 100 mg/mL streptomycin at 37 °C in 95% air—5% CO_2_—. C2C12 myoblasts cell line was obtained from the Department of Molecular Cell Biology (Sungkyunkwan University, Suwon, Republic of Korea). C2C12 myoblasts were maintained as previously described (1). They were cultured in a growth medium (Dulbecco’s Modified Eagle Medium high glucose, Gibco) containing 20% FBS (Gibco), 100 U/mL penicillin, and 100 mg/mL streptomycin at 37 °C in 95% air—5% CO_2_. Differentiation medium contained (Dulbecco’s Modified Eagle Medium high glucose) 1% FBS, 100 U/mL penicillin, and 100 mg/mL streptomycin. The cells were exposed to a single dose of X-ray using an X-ray generation M-150WE (Softex, Tokyo, Japan) at a dose rate of 0.38 Gy/min. For over-expressing PAI-1-GFP protein, the plasmid (PAI-1 cDNA ORF Clone, Human, C-GFPSparkR tag, Sino Biological, HG10296-ACG) was inducted in U87MG cells.

### 2.3. A Literature Review to Find the Relationship between GBM, Radiotherapy, and Fatigue

**Figure 1:**
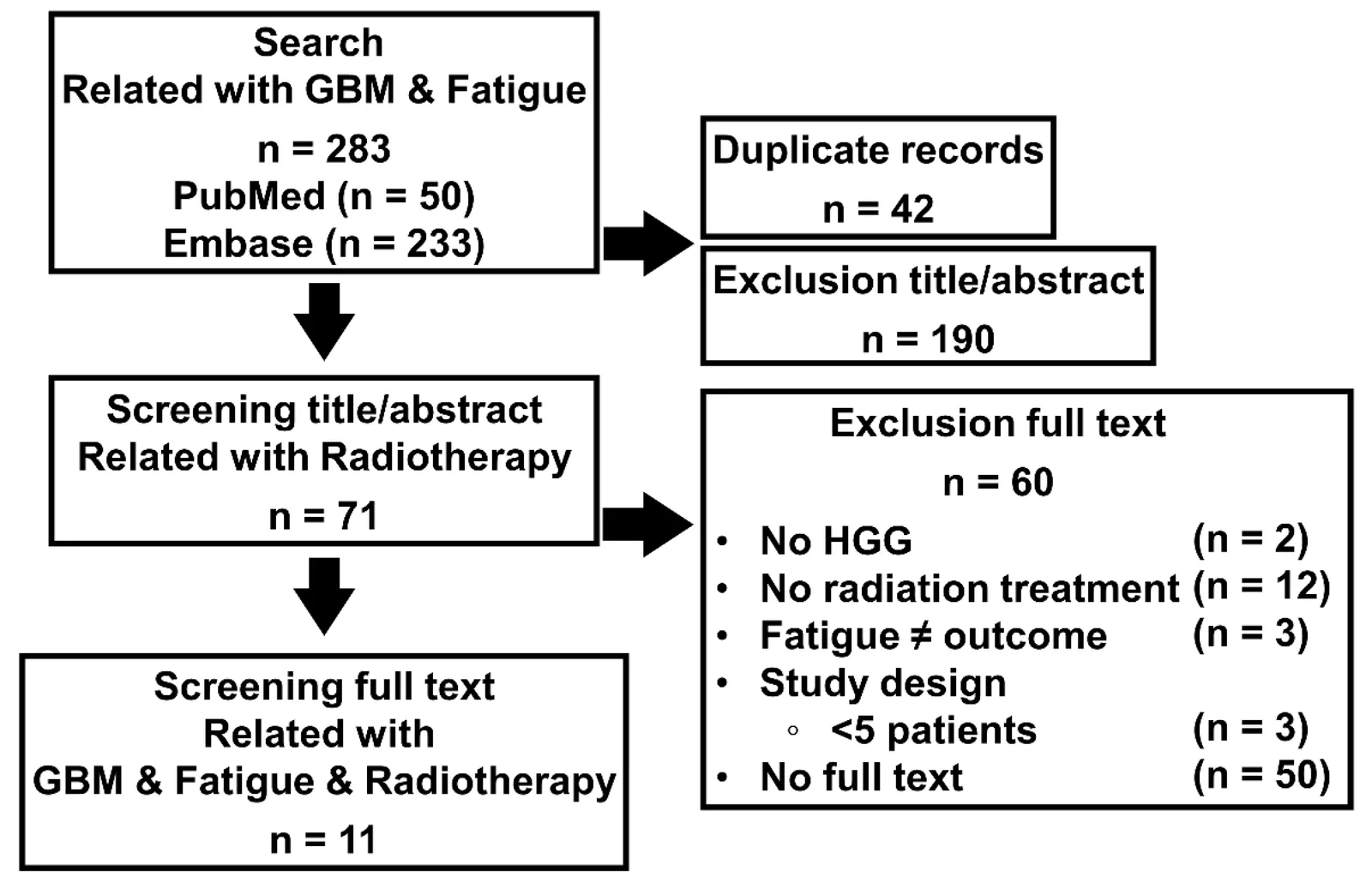
Radiotherapy caused fatigue, a symptom of cachexia in GBM patients. Flowchart of study selection. Various patient-based research was analyzed related to “GBM”, “radiotherapy”, and “fatigue”. The initial search identified 283 articles. After the removal of duplicate studies from the two databases “PubMed” and “Embase”, the titles/abstracts of 241 studies related to radiotherapy, as well as their full text, were screened according to the inclusion criteria. Numerous articles (n = 190) were excluded because no radiation treatment was included. In addition, a relatively large number of articles were found to be abstracts of (-oral-) presentations in various conference meetings (n = 50) and did not include high-grade glioma/glioblastoma patients (n = 2). The final dataset consisted of 11 articles that met all criteria.

A literature review was conducted to find evidence that GBM patients who received radiotherapy were likely to develop cancer cachexia. Data sources were “PubMed” and “Embase”. This paper includes search conditions GBM and fatigue, and detailed search terms are listed in Table S1. A total of 283 related papers were selected as a result of performing an initial search with the relevant search term. Among them, 71 papers were selected, excluding duplicate papers and papers that were incorrectly searched in title or abstract. Here, papers related to radiotherapy were selected. At this time, papers that were not high-grade glioma, papers without radiotherapy, papers without fatigue symptoms, papers with fewer than 5 patients, and papers without full text were excluded. Through this process, 11 related papers were finally selected, and the flow is shown in Figure 1. As a result of the analysis of 11 papers, it was found that 178 of the 520 GBM patients who received radiotherapy experienced 1st–2nd stage fatigue, and 99 patients experienced 3rd–5th stage fatigue.

### 2.4. Animal Care Protocol

12-week-old male BALB/c athymic nude mice (Orient Bio, Seongnam, Korea) were used for the in vivo experiments. The animal protocols were approved by the Institutional Animal Care and Use Committee of Pusan National University (Busan, Korea) (approval number PNU-2021-2972), and experiments were performed under provisions of the National Institutes of Health’s Guide for the Care and Use of Laboratory Animals. The mice were maintained in animal care facilities in a temperature-regulated room (23 ± 1 °C) under a 12 h light/dark cycle and were fed water and standard mouse chow ad libitum.

### 2.5. Orthotopic Xenograft Mouse Model

The U87MG-luc cells were harvested and suspended at a density of 1 × 10^5^ cells/μL in serum-free media. Then, 5 × 10^5^ cells were stereotactically injected into the brain of mice (n = 10 for each group, weight: 18 ± 2 g). 18 days after the injection date, tumor xenografts mice brains were irradiated with 2 Gy daily for 5 days at a dose rate of 600 MU/min using a TrueBeam STx (Varian Medical Systems, Palo Alto, CA, USA). The radiation was delivered by using an 8 mm-diameter collimator. Xenograft growth was monitored by bioluminescent imaging using VISQUE In vivo Smart LF (Vieworks, Anyang, Republic of Korea). At the end of the treatment period, the animals were euthanized, and brain samples, serum, and gastrocnemius were harvested. The non-xenograft mice group was also irradiated at the same dose rate and the same region as the orthotopic xenograft mice model.

### 2.6. Gastrocnemius Isolation and Measurement

The gastrocnemius muscle is in the back part of the lower leg. It runs from its two heads just above the knees to the heel, i.e., three joint muscles (knee, ankle, and subtalar joints). For isolating the gastrocnemius, the mice were sacrificed and the skin of both legs was peeled. We cut the tendon attached to the gastrocnemius using surgical scissors. The isolated gastrocnemius was washed in PBS and the length was measured using a digital vernier caliper.

### 2.7. Hematoxylin and Eosin (H&E) Staining and Immunohistochemistry (IHC)

The mouse brain tissues and skeletal muscle tissues were fixed in formalin, dehydrated, and embedded in paraffin blocks, and the sections were prepared by HistoCore AutoCut (Leica, Deerfield, IL, USA). Next, the sections were cut into 4 μm sections and stained with H&E, following standard procedures. For IHC, sections were treated with 3% hydrogen peroxide/methanol and then with 0.25% pepsin to retrieve antigens. Next, samples were incubated in a blocking solution (Dako, Carpinteria, CA, USA), after which they were incubated at 4 °C overnight with the specific primary antibodies diluted in the antibody diluent (Dako). The sections were subsequently washed with tris buffered saline with 0.1% Tween 20 and then incubated with polymer horseradish peroxidase-conjugated secondary antibody (Dako). A 3,3′-diaminobenzidine substrate chromogen system (Dako) was utilized to detect antibody binding. Stained sections were observed under an Olympus IX71 inverted microscope (Olympus Optical, Tokyo, Japan). The size of muscle fibers can be observed by H&E staining of the gastrocnemius. After taking a picture under a microscope, the cross-sectional area (CSA) of each muscle fiber was measured and compared using the ImageJ program. From 8 mice in each group, 40 photos of H&E staining results were obtained, and the area of 20 randomly selected muscle fibers cross-sectional area was measured for each photo. After being measured, the muscle fiber cross-sectional area was compared with non-treated mice muscle fiber cross-sectional area and analyzed using The Prism 9 software (GraphPad Software, San Diego, CA, USA).

### 2.8. Preparation of Conditioned Media

Cells were plated at a density of 1 × 10^8^ cells/mL in 150-mm culture dishes, incubated for 24 h, and then exposed to 6 Gy of IR. At 24 h after irradiation and transfection of PAI-1 siRNA, cells were washed with PBS three times, then further incubated in serum-free media without antibiotics for 48 h. CM were collected and filtered through a 0.45 μm syringe filter to remove any residual cells. Filtered CM was concentrated 10-fold using a Centricon-10 concentrator (Millipore, Billerica, MA, USA) at 4 °C, then stored at −20 °C. Following CM collection, the number of cells on the dish was determined and the volume of CM used in each experiment was normalized for cell number.

### 2.9. Silver Staining

Expression changes in the secretome of U87MG conditioned mediums were visualized using the silver staining method. The secretomes were separated on 10% polyacrylamide gel and performed silver staining using the silver staining method. Briefly, following electrophoresis, the polyacrylamide gel was placed in fixative solution (5 mL of acetic acid, 20 mL of ethanol, 25 mL of D.W.) at RT for 2 h and then sensitized in sensitizing solution (15 mL ethanol, 2 mL 5% sodium thiosulfate, 1.4 g sodium acetate, 33 mL D.W.) for 30 min to increase the sensitivity and contrast of the staining. We washed them in D.W. twice for 10 min and stained them in staining solution (5 mL 2.5% AgNO3, 20 μL formaldehyde, 45 mL D.W.) for 20 min. We washed them in D.W. twice for 1 min again and developed them in a developing solution (1.25 g Na_2_CO_3_, 10 μL formaldehyde, 50 mL D.W.) until the wanted band appeared. Finally, we stopped the reaction in a stop solution (0.73 g EDTA-Na2·2H2O in 50 mL D.W.) for 10 min.

### 2.10. Exosome Isolation and Characterization

Exosomes were isolated from GBM cell culture supernatants called conditioned medium/media (CM). In brief, CM was collected and differentially centrifuged at 300 × *g* for 10 min, 1000 × *g* for 20 min, and 10,000× *g* for 30 min. Next, the CM was filtered using 0.22 μm filter units (Millex-GP; EMD Millipore, Darmstadt, Germany) and ultracentrifuged at 100,000× *g* for 60 min, and 2 fractions at 4 °C (Beckman Coulter, Brea, CA, USA). After removing the supernatant, the exosome pellets were resuspended in PBS and stored at 4 °C. 

### 2.11. Transmission Electron Microscopy (TEM)

Exosomes were resuspended in PBS and placed onto copper grids at room temperature. The extra solution was removed with filter paper, and the phosphotungstic acid solution was added. Then, the filter paper was used again to remove the excess solution. The copper grids were dried at room temperature for 2 min, and the exosomes were observed with transmission electron microscopy (JEM-1200EX, JEOL Ltd., Tokyo, Japan). This experiment was performed at Pusan National University Hospital.

### 2.12. Peptide Mass Fingerprinting (PMF)

For protein identification by PMF, protein spots were excised, digested with trypsin (Promega, Madison, WI, USA), mixed with a-cyano-4-hydroxycinnamic acid in 50% acetonitrile/0.1% TFA, and subjected to MALDI-TOF analysis (Microflex LRF 20, Bruker Daltonics). Spectra were collected from 300 shots per spectrum over the *m*/*z* range of 600–3000 and calibrated by two-point internal calibration using Trypsin auto-digestion peaks (*m*/*z* 842.5099, 2211.1046). The peak list was generated using Flex Analysis 3.0. The threshold used for peak-picking was as follows: 500 for a minimum resolution of monoisotopic mass, 5 for S/N. The search program MASCOT, developed by Matrixscience, was used for protein identification by PMF. The following parameters were used for the database search: trypsin as the cleaving enzyme, a maximum of one missed cleavage, iodoacetamide (Cys) as a complete modification, oxidation (Met) as a partial modification, monoisotopic masses, and a mass tolerance of ±0.1 Da. The PMF acceptance criteria involve probability scoring.

### 2.13. CM or Exosome Treatment on C2C12 Cell for the Short-Term or Long-Term

For imitating the cancer cachexia condition and verifying the effects on cachexia phenotypes, two separate experiments (short-term and long-term treatments) were performed. Short-term treatment experiments were incubated with CM or exosome for 3 days. Long-term treatment experiments were incubated with CM or exosome for 10 days. Over 10 days of incubation, the C2C12 cell condition had rapidly deteriorated. After treatment, cells were harvested, and performed appropriate experiments.

### 2.14. C2C12 Differentiated Myotube Diameter Measurement

For verifying the effects on cachexia phenotypes in vitro, C2C12 differentiated myotube was stained with MHCI antibody and analyzed by immunofluorescence. Cells were fixed in 4% paraformaldehyde at room temperature for 20 min and permeabilized with 0.5% Triton X-100 for 10 min. Subsequently, cells were rinsed three times with PBS and blocked in blocking buffer (0.1% BSA in PBS) for 30 min. Cells were incubated overnight with the MHCI primary antibodies at 4 °C and washed three times with PBS. After being incubated with DyLight 488-conjugated secondary antibodies (Thermo Scientific), cells were mounted with Fluoroshield Mounting Medium with DAPI (Abcam). Fluorescent images were visualized using a Leica DMi 8 fluorescence microscope (Leica, Wetzlar, Germany). 10 pictures were taken for each sample, and the width of all single myotubes observed per picture was measured using the ImageJ program.

### 2.15. DiO Vibrant Exosome Labeling

For labeling the exosome, the Vibrant DiO cell labeling solution (Invitrogen, V22886) was used. We added 1 μL of 50 nM DiO solution to 50 μL of isolated U87MG-derived exosome. We tapped tubes to mix and incubated for 20 min at 37C in the dark. After 20 min, DiO-labeled exosomes were incubated with C2C12 differentiated myotube for 24 h at 37 °C in the dark. Fluorescent images were visualized using a Leica DMi 8 fluorescence microscope (Leica, Wetzlar, Germany). 

### 2.16. Total RNA Isolation and qRT-PCR

Expression levels of mRNAs were analyzed by real-time qRT–PCR. Total RNA was extracted from C2C12 cells with TRIzol reagent (Invitrogen, Carlsbad, CA, USA) according to the manufacturer’s instructions, after which 1 μg of total RNA was used for cDNA synthesis and Real-time qRT-PCR was performed using an Applied Biosystems StepOne Real-Time PCR System (Applied Biosystems, Foster City, CA, USA). It was performed for 40 cycles of 95 °C for 15 s and 60 °C for 1 min followed by thermal denaturation. The qPCR primers were listed in Table S2. Each sample was assessed in triplicate. Real-time qRT-PCR data were normalized using four housekeeping genes (GAPDH, TBP, RPS3, and eIF3). The mRNA expression data normalized with the geometric mean of the Ct values of four housekeeping genes (GAPDH, TBP, RPS3, and eIF3). The PAI-1 mRNA expression graph normalized with each Ct value was placed in the Supplemental Figures S1–S7.

### 2.17. Western Blot Analysis

After the desired treatments, whole cell lysates were prepared using ProEXTM CETi Lysis Buffer (with protease and phosphatase inhibitors, TransLab, Daejeon, Korea) and the concentrations of protein were determined using a BioRad protein assay kit (BioRad Laboratories, Hercules, CA, USA). The protein samples were subjected to SDS-PAGE, transferred to the 10% nitrocellulose membrane, and then blocked with 5% bovine serum albumin in TBST (10 mM Tris, 100 mM NaCl, and 0.1% Tween 20) for 2 h at room temperature. Next, the membranes were probed with specific primary antibodies at 4 °C overnight and subsequently probed with peroxidase-conjugated secondary antibodies (Santa Cruz Biotechnology, Santa Cruz, CA, USA). The membranes were visualized using an ECL detection system (Roche Applied Science, Indianapolis, IN, USA) with iBright chemi-doc fl000 from Thermo Fisher Scientific (Thermo Fisher Scientific, Cleveland, OH, USA). For each analysis, three biological replicates and three technical replicates were performed. We quantified the vinculin protein level, which was used for reference protein, using imageJ program. Each protein level (STAT3, mTOR, AKT, Atrogin-1, MuRF1, PAI-1) was quantified using the imageJ program and normalized with corresponding vinculin protein levels. The phosphorylated protein levels (p-STAT3, p-mTOR, p-AKT) were quantified using imageJ program and normalized with corresponding unphosphorylated proteins (STAT3, mTOR, AKT). After normalizing the quantified protein levels, each protein level was compared with the first-lane protein levels.

### 2.18. Transwell Co-Culture System

The Transwell co-culture experiment was used the Transwell plate, Corning^®^ 24 mm Transwell^®^ with 0.4 µm Pore Polyester Membrane Insert, Sterile. For the co-culture experiment, C2C12 myoblasts were placed in the upper chamber and incubated with a differentiation medium for differentiation to C2C12 myotube. After differentiation, U87MG cells were placed in a lower chamber and replaced with a serum-free DMEM medium for 10 days (long-term treatment). After 10 days, C2C12 differentiated myotubes were harvested and assessed by western blot analysis.

### 2.19. Statistical Analysis

All numerical data are presented as the means ± standard error of the mean from at least three independent experiments. For quantifications, a two-tailed unpaired Student’s t-test was used for comparing two experimental groups, and one-way ANOVA was applied when needed to compare three or more experimental groups. The log-rank (Mantel–Cox) test was used for statistical analysis of survival. The Prism 9 software (GraphPad Software, San Diego, CA, USA) was used for all statistical analyses. A *p*-value < 0.05 was considered to be statistically significant.

## 3. Results

### 3.1. Radiotherapy Caused Fatigue, a Symptom of Cachexia in GBM Patients

Cachexia is a muscle degenerative disorder in which most patients complain of feeling extreme fatigue (over second-grade fatigue), drowsy, and weight loss, because of muscle loss [1,2,25]. In particular, extreme fatigue is the most common symptom of cancer cachexia and is caused by the wasting of muscles throughout the body, which reduces the patient’s quality of life. For verifying whether GBM patients experienced increased sensations of extreme fatigue post-treatment with radiotherapy, various patient-based research was analyzed related to “GBM”, “radiotherapy”, and “fatigue” from the two databases “PubMed” and “Embase” (Table S1). Following the flowchart works, the final dataset consisted of 11 articles that met all criteria (Figure 1), and these studies are summarized (Table 1). According to our analysis, after treatment with radiotherapy, some patients with GBM experience grades 1 and 2 fatigue, and some patients experience serious fatigue. These findings indicated that radiotherapy-treated GBM patients demonstrate a probability of developing cancer cachexia. Accordingly, our study aimed to identify the major factors involved in the development of cancer-associated cachexia in GBM patients subjected to radiotherapy.

### 3.2. PAI-1 Expression and Secretion Were Elevated in Irradiated GBM Cells and an IR-Induced GBM Cachexia Mouse Model

**Figure 2:**
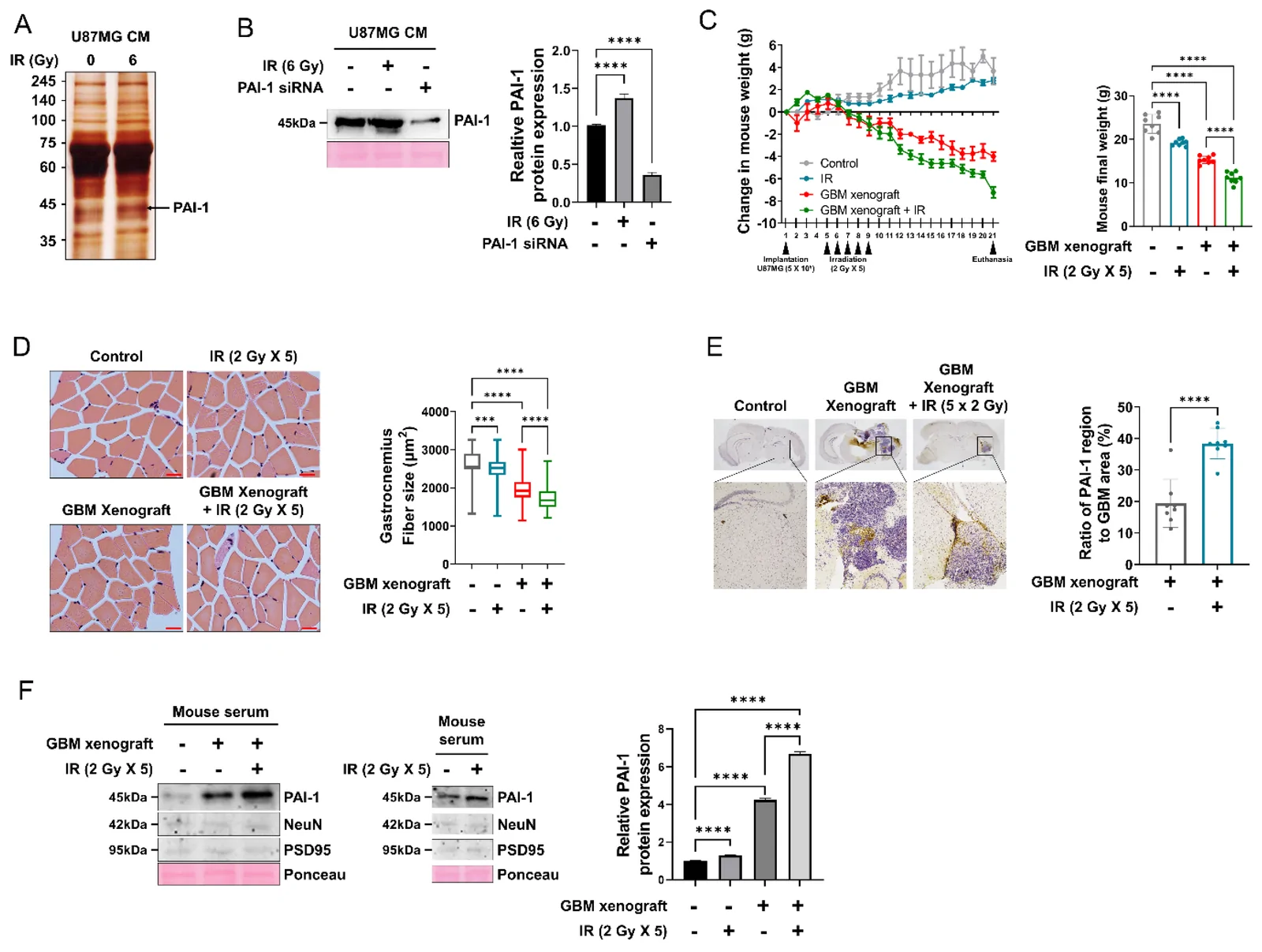
PAI-1 expression and secretion were elevated in irradiated GBM cells and an IR-induced GBM cachexia mouse model. (**A**) The secretome in CM of U87MG cells exposed to 6 Gy was analyzed by silver staining and proteomics using mass spectrometry. The band indicated by an arrowhead corresponds to the PAI-1 protein, which was increased by irradiation. (**B**) Radiation-induced expression levels of the PAI-1 protein in CM of U87MG cells and treated with PAI-1 siRNA were analyzed by western blotting (left panel), and the PAI-1 protein was quantified (right panel). (**C**) Mouse body weight analysis on in vivo in a xenograft mouse model and irradiated xenograft mouse model. Balb/c nude mice were inoculated with 5 × 10^5^ U87MG GBM tumor cells and irradiated 2 Gy daily for 5 days (left panel). The final body weights of the mice were analyzed (right panel). (**D**) Mouse skeletal muscles were stained with hematoxylin and eosin (H&E) to demonstrate the extent of the muscle atrophy. The histological changes were assessed in control, IR, GBM xenograft, GBM xenograft, and irradiated mice. Photomicrographs were taken at 20× magnification. Scale bars, 100 µm. Myofiber cross-sectional area size in gastrocnemius was measured by ImageJ. (**E**) Representative immunohistochemical (IHC) PAI-1 protein staining in mouse brain GBM tumor (left panel). Mouse brain was stained with PAI-1 antibody and DAPI (blue). Scale bar, 100 mm. Occupancy ratio of PAI-1 within the tumor area of GBM xenograft mice (right panel) (**F**) The protein expression of PAI-1, NeuN, and PSD95 in mouse blood serum samples were assessed by western blot (left panel). PAI-1 protein was quantified (right panel). Every in vitro western blot analysis was performed three times. In vivo experiment was performed with 8 mice in each group. Statistical analysis was performed with one-way ANOVA plus Tukey’s multiple comparisons test. *** *p* < 0.005, **** *p* < 0.0001.

Radiotherapy of GBM involves irradiation of a specific tumor region in the brain, which results in the altered release of various factors. As the brain and muscle tissue are spatially quite distant, when the brain is exposed to radiation, the only way for the irradiation-induced alterations in the brain to affect the muscle is through the secretome. It was hypothesized that radiation-induced changes in the secretome of GBM cells are directly transferred to the skeletal muscle cells and induce cancer cachexia. To verify whether cachexia is induced by radiation, radiotherapy was performed in normal mice. As a result of the experiment, it was confirmed that the weight and muscle fiber size of mice decreased after irradiation (Figure 2C,D). To identify the key factors that cause radiation-induced GBM cachexia, the secretome from irradiated U87MG GBM cells mimicking the effects of radiotherapy were analyzed. Conditioned medium (CM) from non-irradiated or irradiated U87MG cells was assessed by a silver-staining assay, and the identities of several proteins were determined by peptide mass spectrometry (MALDI-TOF) with high confidence. Subsequent data analysis revealed various candidates, including PAI-1, significantly elevated expression by irradiation (Figure 2A, Figure S1A). The expression of PAI-1 protein was measured in U87MG CM; secreted levels of PAI-1 were increased in response to radiation in irradiated U87MG cells CM (Figure 2B). To confirm that PAI-1 protein is associated with cachexia, BALB/c nude mice were inoculated with 5 × 10^5^ U87MG GBM tumor cells and irradiated with 2 Gy daily for 5 days to establish in vivo GBM cachexia mouse model. Subsequent observations revealed that the total body weight was not only decreased in the GBM xenograft group but even further reduced in the irradiated GBM xenograft group, significantly (Figure 2C). Furthermore, gastrocnemius muscle size was decreased in the irradiated GBM xenograft group as compared to that in the non-irradiated group (Figure S1B–D). Gastrocnemius muscles were subjected to H&E staining to verify changes in the muscle diameter. H&E staining revealed that the skeletal muscle cross-sectional area (CSA) of irradiated GBM xenograft mice was decreased significantly as compared to that in the non-irradiated GBM xenograft mice (Figure 2D). IHC analysis with mouse brain tissues revealed that both GBM xenograft mouse brain and irradiated GBM xenograft mouse brain expressed high levels of PAI-1 protein in tumor regions. When comparing the proportion of PAI-1 in the tumor regions, it was also confirmed that PAI-1 occupies a larger proportion in the tumor in the GBM xenograft plus IR group. (Figure 2E). Moreover, the secreted PAI-1 levels in mouse serum were shown to be elevated in both GBM xenograft mouse serum and irradiated GBM xenograft mouse serum (Figure 2F). Normal mice serum with irradiation also presented an elevated PAI-1 protein level (Figure 2F). Since there is a possibility that radiation may cause damage to the BBB, the expression of neuronal markers NeuN and PSD95 in mouse serum was checked to determine whether the increase in PAI-1 is due to damage to the BBB. As a result of the experiment, NeuN and PSD95 were detected at very low levels in normal mouse serum and after irradiation rather than hardly detected (Figure 2F). Together, these results indicated that GBM and irradiated GBM induces increased PAI-1 protein expression, which may be involved in the development of GBM cachexia and radiation-induced GBM cachexia.

### 3.3. Muscle Homeostasis Was Disrupted by IR-Induced PAI-1 Secretion from GBM Cells

**Figure 3:**
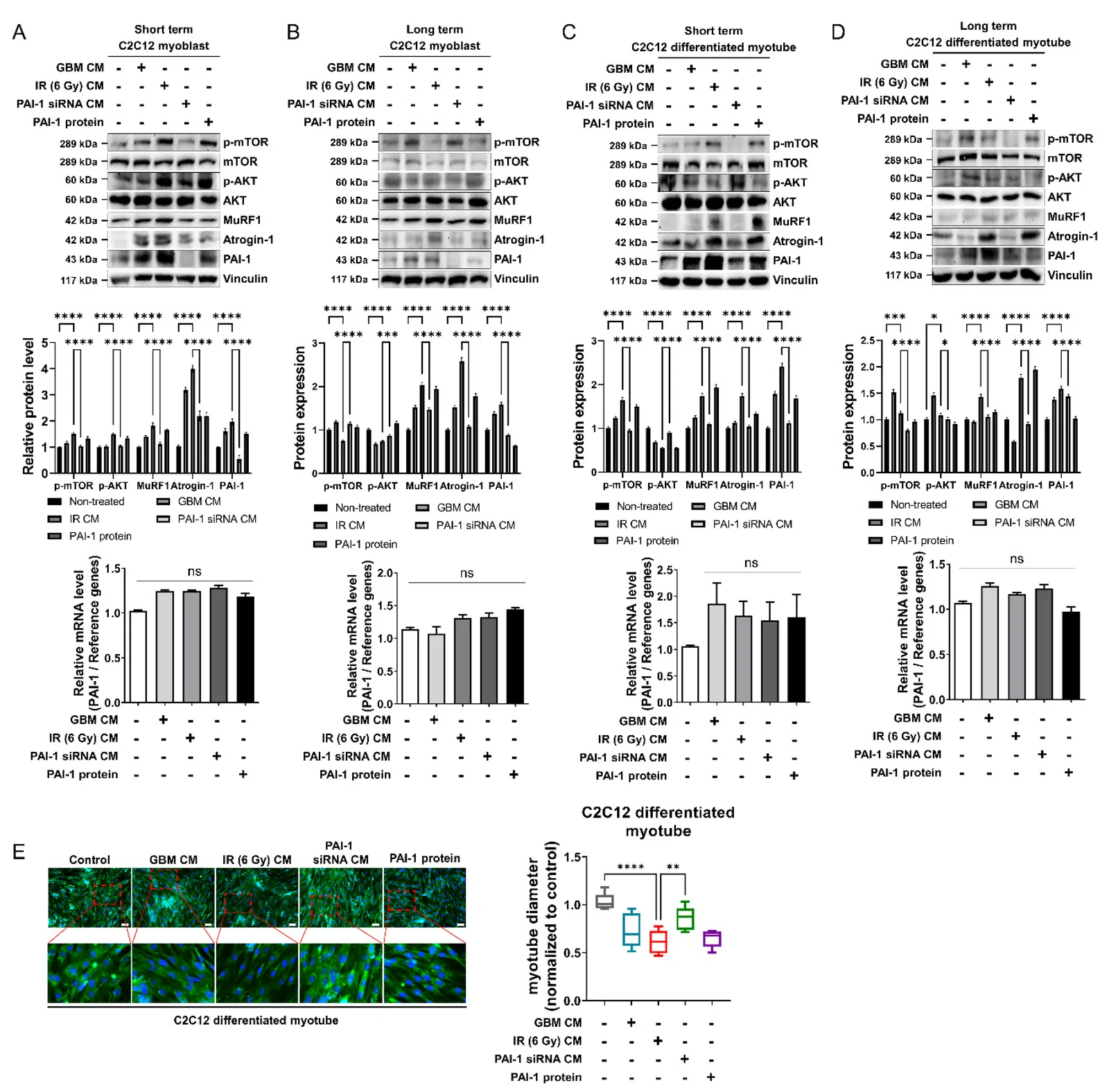
Muscle homeostasis was disrupted by IR-induced PAI-1 secretion from GBM cells. (**A**,**B**) The protein expression of muscle protein synthesis (p-mTOR, p-AKT), muscle protein degradation (MuRF1, Atrogin-1) related proteins, and the PAI-1 protein in the C2C12 myoblast cell treated with U87MG CM in the short-term or long-term were assessed by western blot (upper panel), p-mTOR, p-AKT, MuRF1, Atrogin-1, PAI-1 protein were quantified (middle panel), mRNA expressions change of PAI-1 in C2C12 myoblast cell treated with U87MG CM in the short-term or long-term were assessed by qRT-PCR (lower panel). (**C**,**D**) The protein expression of muscle protein synthesis (p-mTOR, p-AKT), muscle protein degradation (MuRF1, Atrogin-1) related proteins, and the PAI-1 protein in C2C12 differentiated myotube cell treated with the U87MG CM in the short-term or long-term were assessed by western blot (upper panel), p-mTOR, p-AKT, MuRF1, Atrogin-1, PAI-1 protein were quantified (middle panel), mRNA expressions change of PAI-1 in C2C12 differentiated myotube treated with U87MG CM in the short-term or long-term were assessed by qRT-PCR (lower panel). (**E**) C2C12 differentiated myotube cells treated with U87MG CM were stained with MHCI (green) and DAPI (blue) to measure myotube diameter. Scale bar, 10 μm. Statistical analysis was performed with one-way ANOVA plus Tukey’s multiple comparisons test. ns: nonsignificant, * *p* < 0.05, ** *p* < 0.01, *** *p* < 0.005, **** *p* < 0.0001.

Muscle cells maintain a homeostatic mechanism to prevent muscle wasting and maintain muscle mass. However, cachexia is a disease that disrupts muscle homeostasis, which manifests itself in the concomitant symptoms of muscle atrophy (catabolic pathway) and muscle protein synthesis (anabolic pathway) [36]. MAFbx/Atrogin-1 and MuRF1 have been identified as muscle-specific E3 ubiquitin ligases that are highly expressed in skeletal muscles during muscle wasting [37,38]. Muscle protein synthesis is mediated by the activation of the mechanistic target of rapamycin (mTOR) and AKT signaling. Furthermore, multiple studies have reported that phosphorylation of mTOR and AKT is suppressed in cachectic skeletal muscles [39]. To identify the role of secreted PAI-1 induces proteins involved in muscle atrophy in vitro, U87MG CM was used to treat mouse myoblast C2C12 cells and differentiated C2C12 myotubes. Since cachexia is a chronic disease, the short- and long-term treatment effects of U87MG CM were compared to imitate cachexia conditions in vitro. Short-term treatment in C2C12 myoblast cells with U87MG CM (so-called GBM CM) or irradiated U87MG CM (so-called IR CM) elevated protein expression levels of MuRF1 and Atrogin-1 compared with the non-treated group or PAI-1 siRNA treated U87MG CM (so-called siRNA CM) treated group. The GBM CM or IR CM treated group also showed elevated protein expression levels of p-mTOR and p-AKT compared with the non-treated group or siRNA CM treated groups (Figure 3A). These findings indicated that GBM-derived PAI-1, which increased in GBM CM or IR CM, not only induces muscle atrophy but also induces muscle protein synthesis. This can be seen as a muscle homeostasis mechanism. Long-term treatment in C2C12 myoblasts to IR CM was found to imitate cancer cachexia phenotype by resulting in elevated expression levels of MuRF1 and Atrogin-1 while decreasing the levels of p-mTOR and p-AKT compared with GBM CM treated group (Figure 3B). The C2C12 differentiated myotubes were also treated under the same conditions as with the C2C12 myoblasts, resulting in similar MuRF1 and MAFbx/Atrogin-1 expression changes and p-mTOR and p-AKT expression changes compared with C2C12 myoblast cells (Figure 3C,D upper panel, middle panel). Additionally, it was confirmed whether the source of PAI-1 protein responsible for muscle atrophy and decreased muscle protein synthesis is U87MG CM or the muscle cells by comparing the transcriptional levels and protein levels of PAI-1 in C2C12 myoblasts and differentiated C2C12 myotubes. Our results revealed that although the treatment of C2C12 myoblast cells and differentiated C2C12 myotubes with IR CM increased the levels of PAI-1 protein, there was no change in the transcriptional levels of PAI-1 (Figure 3A–D lower panel, Figure S2A–D). This finding implied that the source of elevated PAI-1 protein in muscle cells is not the muscle cells but derived from the GBM cells. Furthermore, the C2C12 differentiated myotubes were assessed for MHCI expression by immunofluorescence (IF) to confirm myotube atrophy. The results showed that treatment with IR CM leads to reduced myotube diameter relative to that seen in the GBM CM and siRNA CM treatment groups (Figure 3E). Overall, these findings indicated that long-term exposure to PAI-1 protein derived from irradiated GBM cells can disrupt muscle homeostasis through increased muscle atrophy and decreased muscle protein synthesis.

### 3.4. Ionizing Radiation-Induced PAI-1 from GBM Cells Was Carried by Exosomes

**Figure 4:**
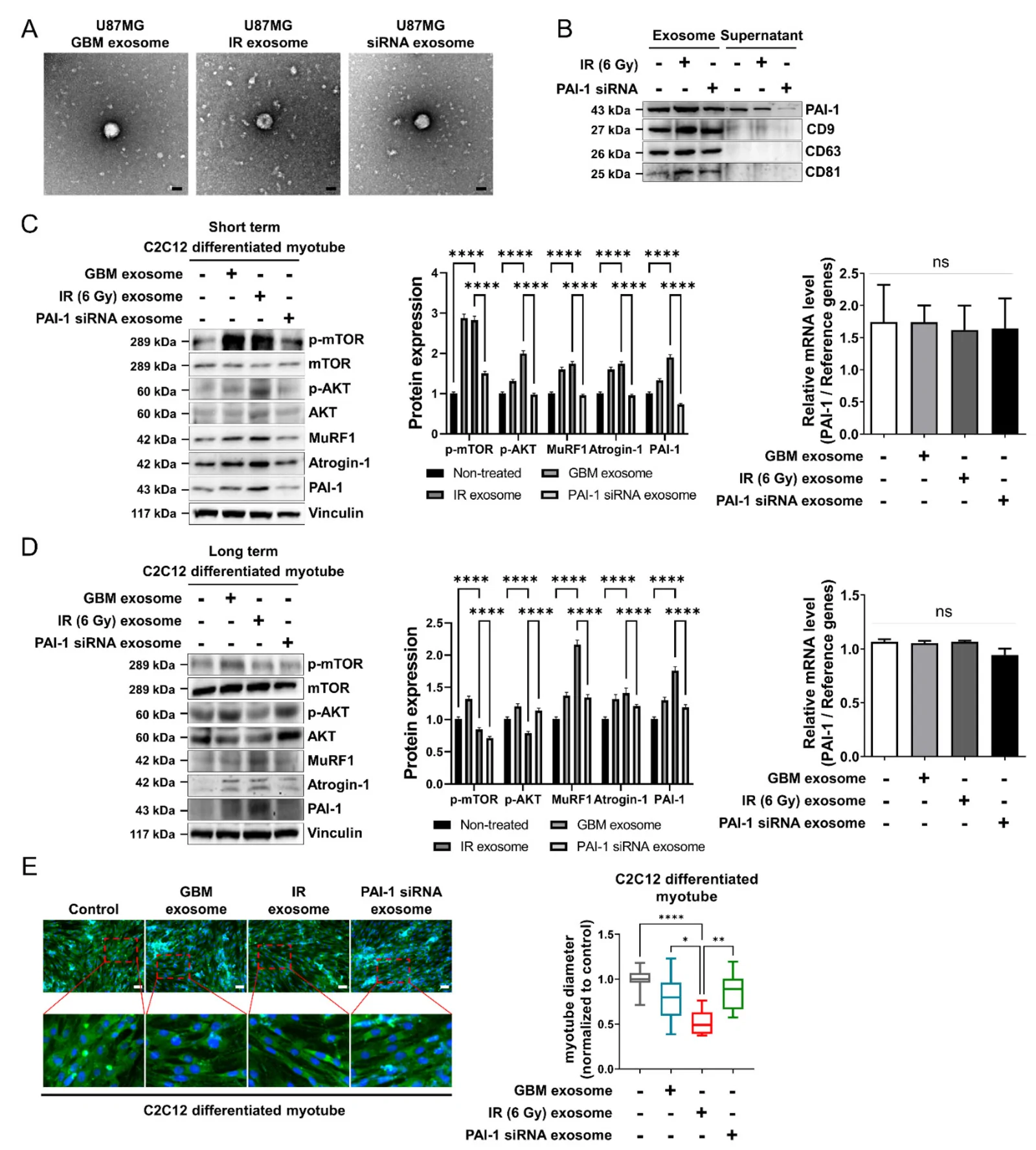
Ionizing radiation-induced PAI-1 from GBM cells was carried by exosomes and induced cachexia. (**A**) Representative images of U87MG exosomes taken by the transmission electron micrograph (TEM), Scale bar, 100 nm. (**B**) PAI-1 protein was highly expressed in exosome fraction. Conditioned medium (CM) from U87MG cells was collected and ultracentrifugation method was performed to isolate exosomes and supernatant. Western blotting was performed to detect the expression of PAI-1 protein and CD9, CD63, CD81 in exosome and supernatant with equal amounts of protein. (**C**,**D**) The protein expression of muscle protein synthesis (p-mTOR, p-AKT), muscle protein degradation (MuRF1, Atrogin-1) related proteins, and the PAI-1 protein in C2C12 differentiated myotube cell treated with the U87MG exosome in the short-term or long-term were assessed by western blot (left panel), p-mTOR, p-AKT, MuRF1, Atrogin-1, PAI-1 protein were quantified (middle panel), mRNA expressions change of PAI-1 in C2C12 differentiated myotube treated with U87MG exosome in the short-term or long-term were assessed by qRT-PCR (right panel). (**E**) C2C12 differentiated myotube cells treated with U87MG exosome were stained with MHCI (green) and DAPI (blue) to measure myotube diameter. Scale bar, 10 μm. Statistical analysis was performed with one-way ANOVA plus Tukey’s multiple comparisons test. ns: nonsignificant, * *p* < 0.05, ** *p* < 0.01, ****p < 0.0001.

Proteins are transported to distant organs by various forms of vesicles or via the bloodstream. Usually, transportation through the bloodstream can result in rapid degradation of the proteins. Conversely, other transport pathways, such as extracellular cargos enveloped in vesicles, have been shown to maintain protein stability [40]. Considering the effects of transportation mechanisms on protein stability, it was hypothesized that the PAI-1 protein, increased upon irradiation of GBM cells, requires a suitable vesicle for safe transportation. Furthermore, as appropriate vesicles for transporting PAI-1 protein, it was hypothesized that exosomes may be involved in enveloping the PAI-1 protein to maintain protein stability during its transportation from GBM cells to distant skeletal muscles. Using the ultracentrifugation method, exosomes from U87MG CM were isolated. Transmission electron microscopy (TEM) showed the presence of 50–100 nm-sized vesicles, which are exosomes (Figure 4A). Further characterization studies revealed the presence of the exosomal protein markers CD9, CD63, and CD81, demonstrating the purity of the isolated exosomes, not in the supernatant (Figure 4B). Moreover, the PAI-1 protein was observed to be present in the purified exosomes but not in the supernatant (Figure 4B). These results indicated that the PAI-1 protein exists in the exosomes derived from U87MG cells. To identify the effect of exosomal PAI-1 on the induction of cachexia in vitro, the C2C12 myoblasts and differentiated C2C12 myotubes were treated with U87MG-derived exosomes for short- and long durations. Our results revealed that short-term treatment of C2C12 myoblasts and C2C12 differentiated myotubes with exosomes derived from irradiated U87MG cells (so-called IR exosome) led to elevated expression levels of MuRF1 and MAFbx/Atrogin-1 as compared with the corresponding expression levels in exosomes derived from PAI-1 siRNA-treated U87MG cells (so-called siRNA exosome). The IR exosome-treated group also showed elevated expression levels of p-mTOR and p-AKT in C2C12 myoblasts and C2C12 differentiated myotubes as compared to the corresponding expression levels in the siRNA exosome-treated group (Figure 4C, Figure S3A). Furthermore, long-term treatment of C2C12 myoblasts and C2C12 differentiated myotubes with IR exosomes were observed to imitate cancer cachexia, such as elevation of MuRF1 and MAFbx/Atrogin-1 expression and decrease in p-mTOR and p-AKT in a fashion similar to the siRNA exosome-treated group as compared with GBM exosome-treated groups (Figure 4D, Figure S3B). Additionally, to confirm whether PAI-1 protein originated from the U87MG-derived exosomes or was synthesized in the muscle cells, PAI-1 mRNA expression levels were tested in C2C12 myoblasts and differentiated C2C12 myotubes and compared with the protein in the same cells. Our results revealed that although the treatment of C2C12 myoblasts and differentiated C2C12 myotubes with IR exosomes led to elevated expression levels of PAI-1 protein, PAI-1 mRNA expression levels remained the same in these cells (Figure 4C–D right panel, Figure S2A–D). Furthermore, C2C12 differentiated myotubes were assessed for MHCI expression by IF to confirm muscle atrophy. Moreover, treatment of C2C12 differentiated myotubes with IR exosomes resulted in reduced myotube diameter relative to that seen in the GBM exosome and siRNA exosome treatment groups (Figure 4E). Overall, these findings indicated that GBM-derived exosomes serve as the source of elevated PAI-1 protein, which plays an important role in the development of cancer cachexia.

### 3.5. Exosomal PAI-1 Was Directly Penetrated via Non-Receptor-Mediated Endocytosis in Muscle Cells

**Figure 5:**
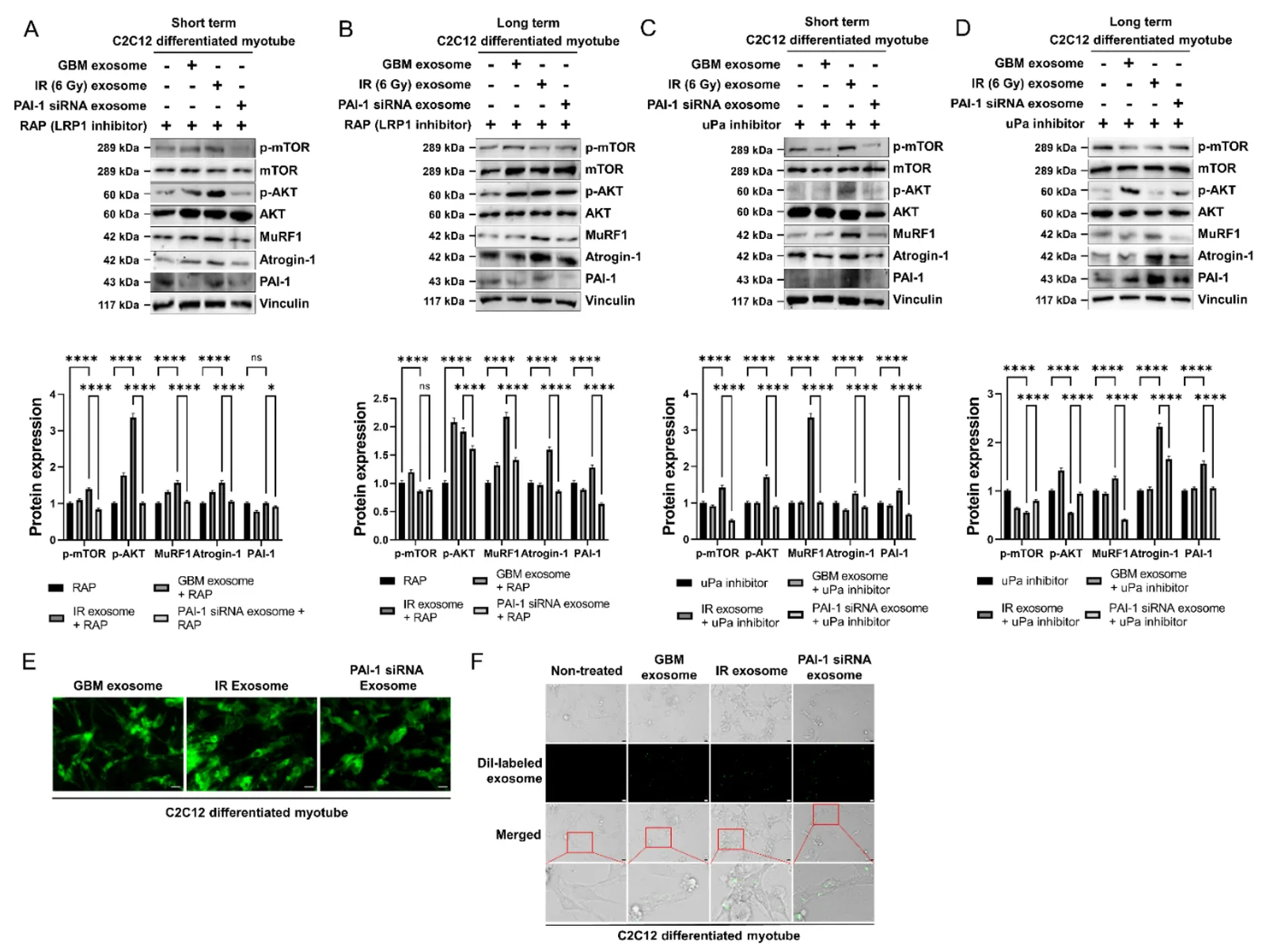
Exosomal PAI-1 was directly penetrated via non-receptor-mediated endocytosis in differentiated myotubes. (**A**,**B**) The protein expression of muscle protein synthesis (p-mTOR, p-AKT), muscle protein degradation (MuRF1, Atrogin-1) related proteins, and the PAI-1 protein in C2C12 differentiated myotube treated with the U87MG exosome and LRP1 inhibitor (RAP) in the short-term or long-term were assessed by western blot (upper panel). p-mTOR, p-AKT, MuRF1, Atrogin-1, PAI-1 protein were quantified (lower panel). (**C**,**D**) The protein expression of muscle protein synthesis (p-mTOR, p-AKT), muscle protein degradation (MuRF1, Atrogin-1) related proteins, and the PAI-1 protein in C2C12 differentiated myotube treated with U87MG exosome and uPA inhibitor in the short-term or long-term were assessed by western blot (upper panel). p-mTOR, p-AKT, MuRF1, Atrogin-1, PAI-1 protein were quantified (lower panel). (**E**) Fluorescence imaging of C2C12 differentiated myotube cells treated with U87MG exosome contained with PAI-1-GFP protein. Scale bar, 10 μm. (**F**) Take DiO-labeled U87MG exosomes and C2C12 differentiated myotube cells to co-culture for 24 h, cellular uptake of exosomes detected by fluorescence microscope Scale bar, 10 μm. Statistical analysis was performed with one-way ANOVA plus Tukey’s multiple comparisons test. ns: nonsignificant, * *p* < 0.05, **** *p* < 0.0001.

Previously documented interaction studies have demonstrated that PAI-1 acts as a substrate for the uPA or LRP-1 receptor [41]. However, in the case of proteins transported by exosomes, it is well known that the cargo proteins are delivered to the target cells directly by fusion with the cell membrane [42]. Accordingly, it was hypothesized that the exosomal PAI-1 protein directly penetrated the muscle cell cytoplasm, without interacting membrane receptors. To verify this hypothesis, the myoblasts and myotubes were treated with either uPA inhibitor or LRP-1 inhibitor RAP along with U87MG CM and U87MG exosomes, and the muscle cachexia phenotype was assessed. The results revealed that both inhibitor treatments showed similar results that did not result in the restoration of cachexia phenotypes (Figure 5A–D, Figure S4A–P). These results indicated that GBM-derived exosomal PAI-1 protein did not bind to its major receptors but was released into the muscle cell cytosol. To further validate the exosomal PAI-1 protein induction, PAI-1 tagged GFP was overexpressed in U87MG cells and exosomes were isolated and added to myotube culture. Another method was performed where the exosome was tagged with a fluorescence DiO tag. IF of exosomal PAI-1-GFP and exosome fluorescence DiO tagging also revealed that exosomal PAI-1 moved into the muscle cell cytosol (Figure 5E–F). Furthermore, these results indicated that the GBM-derived exosomal PAI-1 proteins are directly assimilated into muscle cells and may be involved in several intracellular signaling pathways to induce cancer cachexia.

### 3.6. Exosomal PAI-1 Activated Intracellular STAT3 Pathway in Muscle Cells

**Figure 6:**
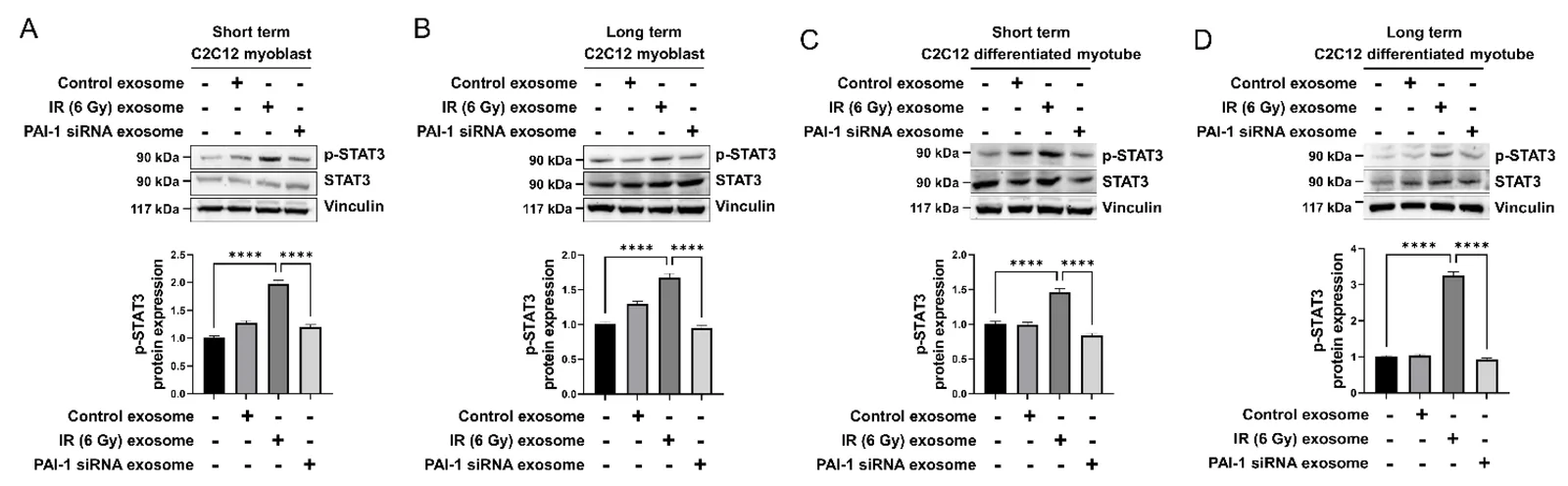
Exosomal PAI-1 activated intracellular STAT3 in differentiated myotubes. (**A**–**D**) The protein expression of phosphorylated STAT3 was assessed by western blot in C2C12 myoblast cell, C2C12 differentiated myotube cell treated with U87MG exosome in the short-term or long-term (upper panel). p-STAT3 level was quantified (lower panel). Statistical analysis was performed with one-way ANOVA plus Tukey’s multiple comparisons test. **** *p* < 0.0001.

The signal transducer and activator of transcription 3 (STAT3) is a transcription factor that mediates the intracellular signaling of several cytokines related to skeletal muscle atrophy. In myofibers, STAT3 promotes muscle wasting by upregulating ubiquitin ligases, leading to muscle degradation [43,44]. A previous study reported that the involvement of STAT3 signaling in muscle wasting syndrome was accompanied by a decrease in muscle protein synthesis in skeletal muscle cells due to elevated levels of phosphorylated STAT3 [45,46]. Accordingly, it was hypothesized that exosome-derived intracellular PAI-1 protein may phosphorylate STAT3 and activate downstream pathways, resulting in decreased muscle protein synthesis and increased muscle atrophy. Subsequent western blot data revealed that treatment with IR CM and IR exosomes markedly increased p-STAT3 expression levels, whereas the same was reduced upon treatment with siRNA CM and siRNA exosomes (Figure 6A–D, Figure S5A–D). Treatment of STAT3 inhibitor presented rescue effects of cachectic phenotypes on C2C12 differentiated myotube (Figure S5E,F). Additionally, C2C12 differentiated myotube incubated with U87MG-derived exosome on Transwell plate also presented cachectic phenotypes (Figure S5G). These results demonstrated that the phenomenon of the phosphorylated STAT3 exists downstream of the intracellular PAI-1 protein signaling pathway and upstream of the muscle cachexia pathway, in addition, it is also accompanied by decreased muscle protein synthesis and increased muscle atrophy.

### 3.7. Pharmacological Inhibition of PAI-1 Rescued IR-Induced GBM Cachexia

**Figure 7:**
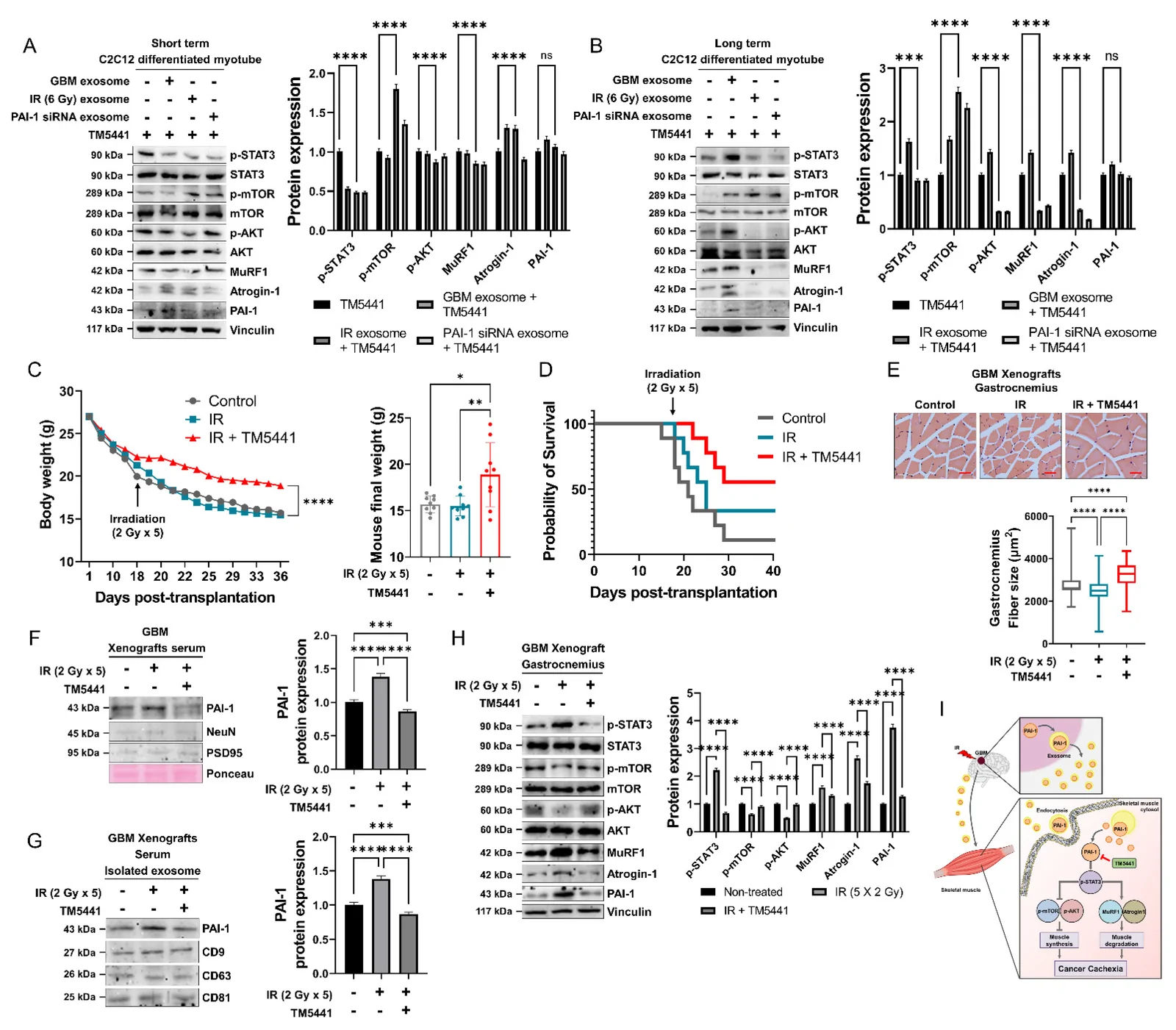
Pharmacological inhibition of PAI-1 rescued IR-induced GBM cachexia. (**A**,**B**) The protein expression of muscle protein synthesis (p-mTOR, p-AKT), muscle protein degradation (MuRF1, Atrogin-1) related proteins, upstream pathway (p-STAT3, STAT3), and PAI-1 protein in C2C12 myoblast cell, C2C12 differentiated myotube treated with U87MG exosome and TM5441, PAI-1 inhibitor in the short-term or long-term were assessed by western blot (left panel). p-STAT3, p-mTOR, p-AKT, MuRF1, Atrogin-1, PAI-1 protein were quantified (right panel). (**C**) Mouse body weights change in three groups of mice; U87MG-luciferase cell xenograft group, U87MG-luciferase cell xenograft plus IR (2 Gy × 5) treated group, U87MG-luciferase cell xenograft plus IR (2 Gy × 5) plus TM5441 treated group. (left panel). The final body weights of the mice were measured (right panel). (**D**) Survival analysis of three groups of mice; U87MG-luciferase cell xenograft group, U87MG-luciferase cell xenograft plus IR (2 Gy × 5) treated group, U87MG-luciferase cell xenograft plus IR (2 Gy × 5) plus TM5441 treated group. Survival analysis was performed by Kaplan-Meier curves and log-rank (Mantel-Cox) test (**E**) Size of gastrocnemius fiber from three groups of mice; U87MG-luciferase cell xenograft group, U87MG-luciferase cell xenograft plus IR (2 Gy × 5) treated group, U87MG-luciferase cell xenograft plus IR (2 Gy × 5 plus TM5441 treated group. Scale bars, 100 µm (upper panel). Myofiber cross-sectional area size in gastrocnemius was measured by ImageJ (lower panel). (**F**) Protein levels of PAI-1, NeuN and PSD95 (controlled by ponceau) were analyzed by western blot in xenografts serum (left panel). PAI-1 protein level was quantified (right panel). (**G**) Protein levels of PAI-1, CD9, CD63 and CD81 were analyzed by western blot in isolated exosomes from xenografts serum (left panel). PAI-1 protein level was quantified (right panel). (**H**) The protein expression of muscle protein synthesis (p-mTOR, p-AKT), muscle protein degradation (MuRF1, Atrogin-1) related proteins, upstream pathway (p-STAT3, STAT3), and PAI-1 protein in Xenografts gastrocnemius treated with IR or TM5441 were assessed by western blot (left panel). p-STAT3, p-mTOR, p-AKT, MuRF1, Atrogin-1, PAI-1 protein was quantified by imageJ (right panel). (**I**) Schematic diagram depicting that exosomal PAI-1 induces ionizing radiation-adaptive GBM cachexia by regulating the intracellular STAT3 pathway. Statistical analysis was performed with one-way ANOVA plus Tukey’s multiple comparisons test. ns: nonsignificant, * *p* < 0.05, ** *p* < 0.01, *** *p* < 0.005, **** *p* < 0.0001.

The PAI-1-specific inhibitor appeared to exhibit therapeutic effects by rescuing muscle atrophy through inhibition of PAI-1, which is a contributory factor of irradiated GBM-induced cachexia. TM5441 has been known as a specific inhibitor of PAI-1 [47,48]. Cell viability assay result revealed that TM5441 did not affect the viability of muscle cells at concentrations under 0.5 μM (Figure S5A). To assess the role of TM5441 in rescuing cachexia phenotype, myoblasts and myotubes were treated with 0.5 μM TM5441 along with U87MG CM or exosome. Treatment with the TM5441 rescued the muscle atrophy phenotype in IR CM or exosome treatment group (Figure 7A,B, Figure S6B–I), and also resulted in decreased activation of STAT3 as compared with the GBM CM- and exosome-treated groups (Figure 7A,B, Figure S6B–I). Overall, these findings indicated that GBM-derived exosomal PAI-1 protein is a critical factor responsible for radiation-induced GBM cachexia, and that TM5441 could prevent muscle cachexia in radiation-induced GBM.

As TM5441 inhibited PAI-1 protein and cachexic effects, TM5441 was verified to exert an in vivo inhibitory effect on radiation-induced GBM cachexia. To verify the TM5441 effects in irradiated GBM cachexia, orthotopic GBM xenografts were prepared by transplanting U87MG-Luc cells into the subventricular zone in the brains of BALB/c nude mice. When tumors reached a similar size (day 7 after U87MG-Luc transplantation), mice were grouped randomly and treated with IR (2 Gy in one day, five times in total) and IR plus TM5441 (20 mg/kg in DMSO, five times in total, oral gavage treatment) (Figure S6J). Body weight was significantly decreased in the control and IR groups. Remarkably, the IR plus TM5441 treated group showed a mild decrease compared with the control and IR group mice (Figure 7C) and remarkably conferred a survival benefit (Figure 7D), but there were no effects on tumor volume (Figure S6K). The results were also determined by fiber size (Figure 7E). As a result of PAI-1 protein analysis in mouse serum and isolated exosome in serum, the level of PAI-1 protein expression was increased in the control group or IR-treated group and was decreased by treatment with TM5441 (Figure 7F,G). Muscle cachectic protein phenotypes were also increased via irradiation and rescued by TM5441 treatment (Figure 7H). Gastrocnemius size and weight also present significantly increased in IR plus TM5441 group compared with the control and IR groups (Figure S6K–M). Collectively, our data supported that PAI-1 protein is a major factor in irradiated GBM cachexia, and inhibition of PAI-1 using TM5441 treatment could be used to rescue radiation-induced GBM cachexia.

Although our study was specifically focused on radiation-induced GBM cachexia, according to existing studies, it is well known that cancer cachexia occurs in a variety of cancer types, including pancreatic, gastric, lung, colorectal, esophageal, and head and neck cancers [16,49]. To investigate whether PAI-1 protein has the potential to induce radiation-induced cachexia in other types of cancer, changes in the expression of PAI-1 protein after irradiation in other cancer cell lines were confirmed. As a result of radiation treatment in cancer cell lines (PanC, AsPC, A549, CaCo2), the upregulation of PAI-1 protein levels was also shown (Figure S6N). Taken together, these data indicate that high levels of exosomal PAI-1 induce IR-induced cancer cachexia, including GBM cachexia, and that of abrogating PAI-1 synergizes with radiation to improve GBM treatment outcomes.

## 4. Discussion

Historically, the treatment of GBM with radiotherapy has demonstrated survival benefits, radiotherapy has been accepted as an adjunct strategy in the management of GBM [50]. As GBM patients have a poor prognosis, limited studies on GBM-associated cachexia exist. However, in a related case report, it was demonstrated that GBM patients also develop cachexia, which impacts their survival rate [51,52]. Additionally, certain studies have reported that radiation therapy induces cachexia in patients with different cancer types [53]. As GBM treatment has improved, along with the increased survival rate of patients, the treatment of GBM cachexia is urgently required to improve treatment efficiency and patients’ quality of life.

Past research studies on GBM patient databases were examined to uncover the possible relationship between GBM treated with radiotherapy and cancer cachexia. Upon scrutinizing the research related to irradiated GBM in data sources, it was focused on increased fatigue in patients as a major symptom of cancer cachexia. It was further confirmed that radiotherapy treated GBM patients possibly experienced fatigue. The results of the present investigation suggested that irradiated GBM patients exhibit a higher possibility of the development of cancer cachexia.

GBM radiotherapy is performed for brain tumor region-specific treatment and it causes alterations in the expression of various factors in GBM tumor cells causing their secretion, and lead to changes in the organ-specific mechanisms [54,55]. In proteomics from irradiated GBM cell-derived CM, PAI-1 was demonstrated to be significantly elevated in irradiated GBM cell secretome, which is enveloped in exosomes for maintain protein stability and participating in cell–cell communication [56]. Additionally, exosome-derived PAI-1 protein did not communicate with receptors, assimilated into the skeletal muscle cytoplasm, resulting in activation of the downstream pathway associated with muscular cachexic pathway. Because the intracellular PAI-1 mechanism is not well defined in the present study, the link between intracellular PAI-1 and the muscular cachexia pathway is missing.

In various cancer types, STAT3 expression was observed to be abrogated specifically in myofibers, and ablation of STAT3 in myofibers prevented muscle loss through the UPS system. STAT3 also regulates muscle stem cell function by antagonizing the self-renewal functions of myofibers to regulate skeletal muscle mass [47,57]. Further investigation is needed to systematically identify the mechanism by which the intracellular PAI-1 downstream pathway connects to muscle atrophy and muscle protein synthesis mechanisms in relation to STAT3. Our results showed that PAI-1 is absorbed into the skeletal muscle cytoplasm and intracellular PAI-1 results in activation of STAT3 phosphorylation, leading to increased muscle atrophy and decreased muscle protein synthesis, thereby resulting in GBM cachexia.

TM5441 has already been demonstrated to exert a beneficial effect of alleviating aging-related muscle fiber atrophy that causes muscle sarcopenia progression [20]. In radiation-induced GBM cachexia, TM5441 co-treatment with radiation rescue cachexic phenotype and improve survival rate. Our in vitro and in vivo findings indicated that a decrease in GBM cachexia not only prevent body weight loss but also rescue the mortality of radiotherapy treated GBM patients.

Controlling GBM cachexia is a crucial factor that has the potential of improving the efficiency of radiotherapy. The results of the present study suggested that increased PAI-1 levels caused by irradiation result in muscle atrophy, finally leading to cachexia. In the present study, we confirmed that PAI-1 secreted from irradiated GBM is delivered to the skeletal muscle cells via exosomes that endocytosed into the skeletal muscle cytoplasm via non-receptor-mediated endocytosis. Chronic intracellular PAI-1 induces STAT3 activation and downstream pathways, including increased muscle atrophy and decreased muscle protein synthesis. Moreover, the obtained results suggested that inhibition of increased PAI-1 using TM5441 is a potential therapeutic strategy to treat GBM cachexia patients subjected to radiotherapy.

Although there is a need for comprehensive active research, our findings indicated that GBM cachexia onset is caused by a side effect of radiotherapy, which is an essential treatment for GBM patients. Additionally, the findings of our study have identified the key factors involved in the development of cachexia in GBM patients after radiotherapy. Most cancer patients do not receive radiation alone but rather go through chemotherapy and radiotherapy after surgery. The reference presented in Table 1 also showed that there were patients who showed extreme fatigue, one of the main symptoms of cancer cachexia after radiotherapy. However, these patients also had received various chemotherapy, and the possibility that fatigue symptoms are caused by multiple drugs cannot be ruled out. Therefore, further studies on the onset of GBM cachexia caused by chemotherapy are also needed. Our results showed that PAI-1 is a key factor involved in the mechanism of cachexia development. The present study provides new insights that we believe will be helpful in controlling the side effects of GBM radiotherapy associated with mortality, by targeting the increase in irradiated GBM-derived exosomal PAI-1 expression, activation of intracellular PAI-1 downstream STAT3 pathway, and disruption of muscle homeostasis in skeletal muscles.

## 5. Conclusions

In this study, we identified PAI-1 as a critical inducer of GBM cachexia. We demonstrated that PAI-1 expression was increased after IR and secreted through exosome. Cachexia is a muscular disease where the inducing factor moves from the brain to the muscle and affects muscle homeostasis. In muscle homeostasis, mTOR and AKT, which are involved in muscle growth, and Atrogin-1 and MuRF1, which are involved in muscle loss, interact at the same time. Exosomal PAI-1 moves to the muscle and endocytosis in muscle cytoplasm; chronic exposure to PAI-1 induces STAT3 activation and leads to muscle homeostasis disruption. Importantly, our in vivo data demonstrate that the PAI-1 effects decrease the muscle fiber diameter and adversely affect the survival rate. The TM5441, PAI-1 inhibitor, blocked the action of PAI-1 in the muscle, alleviated the decrease in muscle fiber, and improved the survival rate. PAI-1 inhibition with TM5441 or other drugs for radiotherapy-treated GBM cachexia patients might improve their survival and life quality.

## Figures and Tables

**Table 1:** Main study characteristics of the included studies.

Article References	GBM Patients (n)	Age Mean	Type of Treatment	Fatigue (Outcome)
[26]	24	58 (36–73)	RT, CT	1st–2nd 8 (33%), 3rd 1 (4%)
[27]	15	57 (39–67)	RT, CT, S	1st–2nd 8 (53%), 3rd 1 (6.7%)
[27]	33	56	RT, CT	1st–2nd 6 (20%)
[28]	25	54 (30–74)	RT, CT, S	Side effect
[29]	36	53.5 (34–68)	RT, CT	1st–2nd 15 (41.6%), 3rd 1 (2.7%)
[30]	48	55 (25–75)	RT, CT, S	1st–2nd 8 (12.9%)
[31]	12	36.5 (26–63)	RT	1st–2nd 11 (91.7%)
[32]	54	57.1	RT, CT, S	1st–2nd 23 (42.6%), 3rd 2 (3.7%)
[33]	125	56.2 (19–80)	RT, CT	4th 2 (1.6%)
[34]	23	48 (20–71)	RT, CT	Side effect
[35]	21	55 (30–76)	RT, CT	1–2nd 16 (73%)

RT; radiotherapy, CT; chemotherapy, S; surgery.

## Data Availability

All data generated or analyzed during this study are included in this published article and can be reused only with the authors’ permission.

## Supplementary Materials

The following supporting information can be downloaded at: https://www.mdpi.com/article/10.3390/cells11193102/s1, Figure S1: Proteomics analysis of GBM cell conditioned media and GBM cachexia phenotypes induced by IR; Figure S2: IR induces upregulation of PAI-1 secreted from GBM cells induce cachexia phenotype in C2C12 myoblasts and differentiated myotubes; Figure S3: IR induces upregulation of PAI-1 secreted from GBM cells induce cachexia phenotype in myoblasts and differentiated myotubes; Figure S4: Secreted PAI-1 and exosomal PAI-1 was directly penetrated via non-receptor-mediated endocytosis in myoblasts and differentiated myotubes; Figure S5: Secreted PAI-1 and exosomal PAI-1 activated intracellular STAT3 in myoblasts and differentiated myotubes; Figure S6: Pharmacological inhibition of PAI-1 rescued IR-induced GBM cachexia; Figure S7: Replicate data of western blot analysis; Table S1: Search strategy; Table S2: Primers for determining levels of gene expression.

## References

[1] Baracos V.E., Martin L., Korc M., Guttridge D.C., Fearon K.C.H. (2018). Cancer-associated cachexia. Nat. Reviews. Dis. Prim..

[2] Cui P., Shao W., Huang C., Wu C.J., Jiang B., Lin D. (2019). Metabolic derangements of skeletal muscle from a murine model of glioma cachexia. Skelet. Muscle.

[3] Louis D.N., Perry A., Wesseling P., Brat D.J., Cree I.A., Figarella-Branger D., Hawkins C., Ng H.K., Pfister S.M., Reifenberger G. (2021). The 2021 WHO Classification of Tumors of the Central Nervous System: A summary. Neuro-Oncology.

[4] Komori T. (2022). The 2021 WHO classification of tumors, 5th edition, central nervous system tumors: The 10 basic principles. Brain Tumor Pathol..

[5] Barani I.J., Larson D.A. (2015). Radiation therapy of glioblastoma. Cancer Treat. Res..

[6] Griffith J.L., Hochberg F.H. (1988). Anorexia and weight loss in glioma patients. Psychosomatics.

[7] van Coevorden-van Loon E.M.P., Coomans M.B., Heijenbrok-Kal M.H., Ribbers G.M., van den Bent M.J. (2017). Fatigue in patients with low grade glioma: Systematic evaluation of assessment and prevalence. J. Neuro-Oncol..

[8] Wu B., Liu W., Zhu H., Feng H., Liu J. (2011). Primary glioblastoma of the cerebellopontine angle in adults. J. Neurosurg..

[9] Wende T., Kasper J., Prasse G., Glass Ä., Kriesen T., Freiman T.M., Meixensberger J., Henker C. (2021). Newly Diagnosed IDH-Wildtype Glioblastoma and Temporal Muscle Thickness: A Multicenter Analysis. Cancers.

[10] Grossberg A.J., Mohamed A.S., Fuller C.D. (2016). Cachexia in Radiotherapy-Treated Patients With Head and Neck Cancer—Reply. JAMA Oncol..

[11] Lees J. (1999). Incidence of weight loss in head and neck cancer patients on commencing radiotherapy treatment at a regional oncology centre. Eur. J. Cancer Care.

[12] Simone C.B. (2019). Cancer cachexia: Definitions, outcomes, and treatments. Ann. Palliat. Med..

[13] Lee J., Lin J.B., Chen T.C., Jan Y.T., Sun F.J., Chen Y.J., Wu M.H. (2021). Progressive Skeletal Muscle Loss After Surgery and Adjuvant Radiotherapy Impact Survival Outcomes in Patients With Early Stage Cervical Cancer. Front. Nutr..

[14] Brierley D.I., Harman J.R., Giallourou N., Leishman E., Roashan A.E., Mellows B.A.D., Bradshaw H.B., Swann J.R., Patel K., Whalley B.J. (2019). Chemotherapy-induced cachexia dysregulates hypothalamic and systemic lipoamines and is attenuated by cannabigerol. J. Cachexia Sarcopenia Muscle.

[15] Argilés J.M., López-Soriano F.J., Stemmler B., Busquets S. (2019). Therapeutic strategies against cancer cachexia. Eur. J. Transl. Myol..

[16] Aoyagi T., Terracina K.P., Raza A., Matsubara H., Takabe K. (2015). Cancer cachexia, mechanism and treatment. World J. Gastrointest. Oncol..

[17] Roeland E.J., Bohlke K., Baracos V.E., Bruera E., Del Fabbro E., Dixon S., Fallon M., Herrstedt J., Lau H., Platek M. (2020). Management of Cancer Cachexia: ASCO Guideline. J. Clin. Oncol..

[18] Sulima S.O., De Keersmaecker K. (2017). Ribosomal proteins: A novel class of oncogenic drivers. Oncotarget.

[19] Werle B., Kotzsch M., Lah T.T., Kos J., Gabrijelcic-Geiger D., Spiess E., Schirren J., Ebert W., Fiehn W., Luther T. (2004). Cathepsin B, plasminogenactivator-inhibitor (PAI-1) and plasminogenactivator-receptor (uPAR) are prognostic factors for patients with non-small cell lung cancer. Anticancer. Res..

[20] Naderi J., Bernreuther C., Grabinski N., Putman C.T., Henkel B., Bell G., Glatzel M., Sultan K.R. (2009). Plasminogen activator inhibitor type 1 up-regulation is associated with skeletal muscle atrophy and associated fibrosis. Am. J. Pathol..

[21] Koh T.J., Bryer S.C., Pucci A.M., Sisson T.H. (2005). Mice deficient in plasminogen activator inhibitor-1 have improved skeletal muscle regeneration. Am. J. Physiology. Cell Physiol..

[22] Krause M.P., Moradi J., Nissar A.A., Riddell M.C., Hawke T.J. (2011). Inhibition of plasminogen activator inhibitor-1 restores skeletal muscle regeneration in untreated type 1 diabetic mice. Diabetes.

[23] Luan X., Sansanaphongpricha K., Myers I., Chen H., Yuan H., Sun D. (2017). Engineering exosomes as refined biological nanoplatforms for drug delivery. Acta Pharmacol. Sin..

[24] Gurung S., Perocheau D., Touramanidou L., Baruteau J. (2021). The exosome journey: From biogenesis to uptake and intracellular signalling. Cell Commun. Signal. CCS.

[25] Fearon K., Strasser F., Anker S.D., Bosaeus I., Bruera E., Fainsinger R.L., Jatoi A., Loprinzi C., MacDonald N., Mantovani G. (2011). Definition and classification of cancer cachexia: An international consensus. Lancet. Oncol..

[26] Guan Y., Wang C., Zhu H., Li J., Xu W., Sun L., Pan L., Dai J., Wang Y., Wang E. (2020). Hypofractionated Radiosurgery Plus Bevacizumab for Locally Recurrent Brain Metastasis with Previously High-Dose Irradiation. World Neurosurg..

[27] Korshoej A.R., Lukacova S., Lassen-Ramshad Y., Rahbek C., Severinsen K.E., Guldberg T.L., Mikic N., Jensen M.H., Cortnum S.O.S., von Oettingen G. (2020). OptimalTTF-1: Enhancing tumor treating fields therapy with skull remodeling surgery. A clinical phase I trial in adult recurrent glioblastoma. Neuro-Oncol. Adv..

[28] Møller S., Munck Af Rosenschöld P., Costa J., Law I., Poulsen H.S., Engelholm S.A., Engelholm S. (2017). Toxicity and efficacy of re-irradiation of high-grade glioma in a phase I dose- and volume escalation trial. Radiother. Oncol. J. Eur. Soc. Ther. Radiol. Oncol..

[29] Sahebjam S., Forsyth P.A., Tran N.D., Arrington J.A., Macaulay R., Etame A.B., Walko C.M., Boyle T., Peguero E.N., Jaglal M. (2021). Hypofractionated stereotactic re-irradiation with pembrolizumab and bevacizumab in patients with recurrent high-grade gliomas: Results from a phase I study. Neuro-Oncology.

[30] Mantica M., Pritchard A., Lieberman F., Drappatz J. (2018). Retrospective study of nivolumab for patients with recurrent high grade gliomas. J. Neuro-Oncol..

[31] Adeberg S., Harrabi S.B., Bougatf N., Bernhardt D., Rieber J., Koerber S.A., Syed M., Sprave T., Mohr A., Abdollahi A. (2016). Intensity-modulated proton therapy, volumetric-modulated arc therapy, and 3D conformal radiotherapy in anaplastic astrocytoma and glioblastoma: A dosimetric comparison. Strahlenther. Onkol..

[32] Soffietti R., Trevisan E., Bertero L., Cassoni P., Morra I., Fabrini M.G., Pasqualetti F., Lolli I., Castiglione A., Ciccone G. (2014). Bevacizumab and fotemustine for recurrent glioblastoma: A phase II study of AINO (Italian Association of Neuro-Oncology). J. Neuro-Oncol..

[33] Vredenburgh J.J., Desjardins A., Kirkpatrick J.P., Reardon D.A., Peters K.B., Herndon J.E., Marcello J., Bailey L., Threatt S., Sampson J. (2012). Addition of bevacizumab to standard radiation therapy and daily temozolomide is associated with minimal toxicity in newly diagnosed glioblastoma multiforme. Int. J. Radiat. Oncol. Biol. Phys..

[34] Hegarty T.J., Thornton A.F., Diaz R.F., Chandler W.F., Ensminger W.D., Junck L., Page M.A., Gebarski S.S., Hood T.W., Stetson P.L. (1990). Intra-arterial bromodeoxyuridine radiosensitization of malignant gliomas. Int. J. Radiat. Oncol. Biol. Phys..

[35] Vogelbaum M.A., Sampson J.H., Kunwar S., Chang S.M., Shaffrey M., Asher A.L., Lang F.F., Croteau D., Parker K., Grahn A.Y. (2007). Convection-enhanced delivery of cintredekin besudotox (interleukin-13-PE38QQR) followed by radiation therapy with and without temozolomide in newly diagnosed malignant gliomas: Phase 1 study of final safety results. Neurosurgery.

[36] Penna F., Ballarò R., Beltrà M., De Lucia S., García Castillo L., Costelli P. (2019). The Skeletal Muscle as an Active Player Against Cancer Cachexia. Front. Physiol..

[37] Yuan L., Han J., Meng Q., Xi Q., Zhuang Q., Jiang Y., Han Y., Zhang B., Fang J., Wu G. (2015). Muscle-specific E3 ubiquitin ligases are involved in muscle atrophy of cancer cachexia: An in vitro and in vivo study. Oncol. Rep..

[38] Castillero E., Alamdari N., Lecker S.H., Hasselgren P.O. (2013). Suppression of atrogin-1 and MuRF1 prevents dexamethasone-induced atrophy of cultured myotubes. Metab. Clin. Exp..

[39] White J.P., Puppa M.J., Gao S., Sato S., Welle S.L., Carson J.A. (2013). Muscle mTORC1 suppression by IL-6 during cancer cachexia: A role for AMPK. Am. J. Physiology. Endocrinol. Metab..

[40] Elsharkasy O.M., Nordin J.Z., Hagey D.W., de Jong O.G., Schiffelers R.M., Andaloussi S.E., Vader P. (2020). Extracellular vesicles as drug delivery systems: Why and how?. Adv. Drug Deliv. Rev..

[41] Bres E.E., Faissner A. (2019). Low Density Receptor-Related Protein 1 Interactions With the Extracellular Matrix: More Than Meets the Eye. Front. Cell Dev. Biol..

[42] Hu Q., Su H., Li J., Lyon C., Tang W., Wan M., Hu T.Y. (2020). Clinical applications of exosome membrane proteins. Precis. Clin. Med..

[43] Sala D., Sacco A. (2016). Signal transducer and activator of transcription 3 signaling as a potential target to treat muscle wasting diseases. Curr. Opin. Clin. Nutr. Metab. Care.

[44] Kawao N., Ishida M., Kaji H. (2019). Roles of leptin in the recovery of muscle and bone by reloading after mechanical unloading in high fat diet-fed obese mice. PLoS ONE.

[45] Gopinath S.D. (2017). Inhibition of Stat3 signaling ameliorates atrophy of the soleus muscles in mice lacking the vitamin D receptor. Skelet. Muscle.

[46] Silva K.A., Dong J., Dong Y., Dong Y., Schor N., Tweardy D.J., Zhang L., Mitch W.E. (2015). Inhibition of Stat3 activation suppresses caspase-3 and the ubiquitin-proteasome system, leading to preservation of muscle mass in cancer cachexia. J. Biol. Chem..

[47] Lang S.Q., Lang W.H., Yu H.Y., Wang L. (2020). Metabolic activation of TM5441 in vitro and in vivo: Formation of reactive metabolites and human enzymes involved. Eur. J. Pharm. Sci..

[48] Boe A.E., Eren M., Murphy S.B., Kamide C.E., Ichimura A., Terry D., McAnally D., Smith L.H., Miyata T., Vaughan D.E. (2013). Plasminogen activator inhibitor-1 antagonist TM5441 attenuates Nω-nitro-L-arginine methyl ester-induced hypertension and vascular senescence. Circulation.

[49] Dhanapal R., Saraswathi T., Govind R.N. (2011). Cancer cachexia. J. Oral Maxillofac. Pathol. JOMFP.

[50] Mann J., Ramakrishna R., Magge R., Wernicke A.G. (2017). Advances in Radiotherapy for Glioblastoma. Front. Neurol..

[51] Elmaci İ., Altinoz M.A. (2016). A Metabolic Inhibitory Cocktail for Grave Cancers: Metformin, Pioglitazone and Lithium Combination in Treatment of Pancreatic Cancer and Glioblastoma Multiforme. Biochem. Genet..

[52] Yee P.P., Wei Y., Kim S.Y., Lu T., Chih S.Y., Lawson C., Tang M., Liu Z., Anderson B., Thamburaj K. (2020). Neutrophil-induced ferroptosis promotes tumor necrosis in glioblastoma progression. Nat. Commun..

[53] Mazzola R., Fiorentino A., Alongi F. (2016). Cachexia in Radiotherapy-Treated Patients With Head and Neck Cancer: A Phenomenon That Should Be Investigated. JAMA Oncol..

[54] Ramakrishnan V., Xu B., Akers J., Nguyen T., Ma J., Dhawan S., Ning J., Mao Y., Hua W., Kokkoli E. (2020). Radiation-induced extracellular vesicle (EV) release of miR-603 promotes IGF1-mediated stem cell state in glioblastomas. EBioMedicine.

[55] Shin E., Lee S., Kang H., Kim J., Kim K., Youn H., Jin Y.W., Seo S., Youn B. (2020). Organ-Specific Effects of Low Dose Radiation Exposure: A Comprehensive Review. Front. Genet..

[56] Doyle L.M., Wang M.Z. (2019). Overview of Extracellular Vesicles, Their Origin, Composition, Purpose, and Methods for Exosome Isolation and Analysis. Cells.

[57] Zhang L., Pan J., Dong Y., Tweardy D.J., Dong Y., Garibotto G., Mitch W.E. (2013). Stat3 activation links a C/EBPδ to myostatin pathway to stimulate loss of muscle mass. Cell Metab..

