# Correction: Shin et al. Exosomal Plasminogen Activator Inhibitor-1 Induces Ionizing Radiation-Adaptive Glioblastoma Cachexia. *Cells* 2022, *11*, 3102

**DOI:** 10.3390/cells15161488

**Published:** 2026-08-19

**Authors:** Eunguk Shin, Hyunkoo Kang, Haksoo Lee, Sungmin Lee, Jaewan Jeon, Kimoon Seong, Hyesook Youn, Buhyun Youn

**Affiliations:** 1 Department of Integrated Biological Science, Pusan National University, Busan 46241, Republic of Korea; 2 Department of Radiation Oncology, Haeundae Paik Hospital, Inje University College of Medicine, Busan 48108, Republic of Korea; 3 Laboratory of Biological Dosimetry, National Radiation Emergency Medical Center (NREMC), Korea Institute of Radiological and Medical Sciences (KIRAMS), Seoul 01812, Republic of Korea; 4 Department of Integrative Bioscience and Biotechnology, Sejong University, Seoul 05006, Republic of Korea; 5 Department of Biological Sciences, Pusan National University, Busan 46241, Republic of Korea

In the original publication [1], there were mistakes in the assembly of several Western blot panels in Supplementary Figures S4, S6, and S7. Specifically, the bands for PAI-1 in Figure S4F appeared highly similar to those for Atrogin-1 in Figure S4K; mTOR bands showed similarities between Figures S4I and S6D, as well as in Figure S6F,G; the band for Atrogin-1 in Figure S4F appeared to be flipped relative to MuRF1 in Figure S4G; and similarities were observed between p-AKT bands in Figure S7F and p-mTOR in Figure S7G, in addition to AKT bands in Figure S7D,E. These errors occurred unintentionally during the final figure assembly and file organization of the large volume of supplementary data that was newly added during the revision process to address reviewer comments.

The corrected versions of the affected Supplementary Figures have been prepared based on the original raw data and are provided in the attached corrected Supplementary Material file. The authors state that the scientific conclusions are unaffected. This correction was approved by the Academic Editor. The original publication has also been updated.
