# Supplementary material for: Exosomal Plasminogen Activator Inhibitor-1 Induces Ionizing Radiation-Adaptive Glioblastoma Cachexia
Source: Cells. 2022 Oct 1;11(19):3102. doi: 10.3390/cells11193102 (PMC9564109; doi:10.3390/cells11193102)
Supplement: Supplementary file 1 [file cells-11-03102-s001.zip › cells-1843109-supplementary.pdf]

**Supplemental Tables**

**Table S1. Search strategy**

**Table S2. Primers for determining levels of gene expression**

**Supplemental Figures**

**Figure S1. Proteomics analysis of GBM cell conditioned media and GBM cachexia phenotypes induced by IR**

**Figure S2. IR induces upregulation of PAI-1 secreted from GBM cells induce cachexia phenotype in C2C12 myoblasts and differentiated myotubes**

**Figure S3. IR induces upregulation of PAI-1 secreted from GBM cells induce cachexia phenotype in myoblasts and differentiated myotubes**

**Figure S4. Secreted PAI-1 and exosomal PAI-1 was directly penetrated via non-receptor-mediated endocytosis in myoblasts and differentiated myotubes**

**Figure S5. Secreted PAI-1 and exosomal PAI-1 activated intracellular STAT3 in myoblasts and differentiated myotubes**

**Figure S6. Pharmacological inhibition of PAI-1 rescued IR-induced GBM cachexia**

**Figure S7. Replicate data of western blot analysis**

**Table S1. Search strategy**

| Data source | Search terms |
|---|---|
| Pubmed | ((Glioblastoma[mh] OR glioblastoma*[tiab] OR GBM*[tiab] OR gbm*[tiab]) AND (high grade*[tiab] OR grade 4*[tiab] OR grade4*[tiab] OR grade IV*[tiab]) AND (fatigue*[tiab])) |
| Embase | 'glioblastoma'/exp OR glioblastoma OR 'GBM'/exp OR glioblastoma*:ab,ti OR Glioblastoma*:ab,ti OR GBM*:ab,ti OR gbm*:ab,ti AND ('high grade':ab,ti OR 'grade iv':ab,ti OR 'grade 4':ab,ti OR 'grade4':ab,ti) AND fatigue*:ab,ti |

**Table S2. Primers for determining levels of gene expression.**

| Gene | Forward primer (5′->3′) | Reverse primer (5′->3′) |
|---|---|---|
| PAI-1 | TCGTCCAGCGGGATCTGA | GTGCTCCGGAACAGCCTG |
| GAPDH | ATGACATCAAGAAGGTGGTG | CATACCAGGAAATGAGCTTG |
| TBP | GAGCTGTGATGTGAAGTTTCC | TCTGGGTTTGATCATTCTGTAG |
| RPS3 | ATTCACTGGCTGACCGGATG | GTGCCAAGGGTTGTGAGGTC |
| eIF3 | CCGCCATCGCTACTGTCTCC | CCTCTTGCGCTCCTGTTCACT |

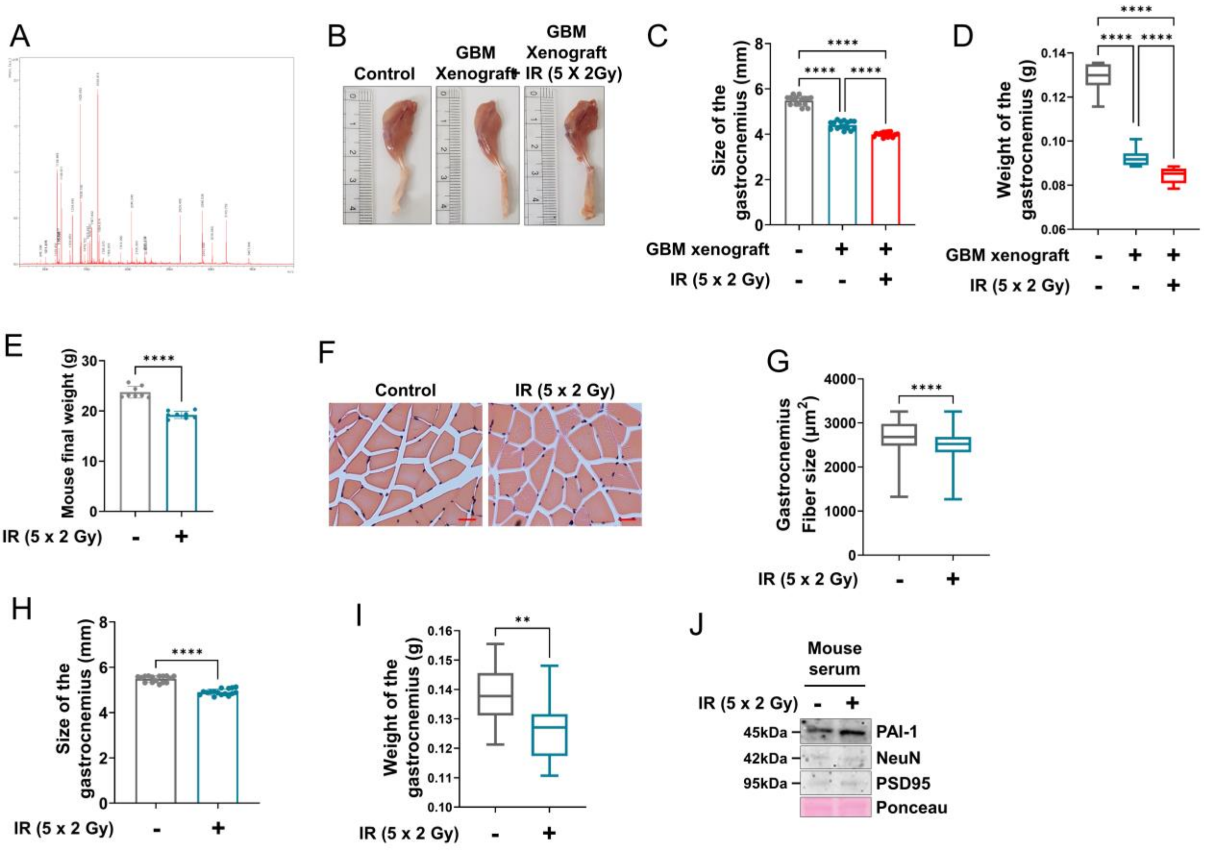


**Figure S1. Proteomics analysis of GBM cell conditioned media and GBM cachexia phenotypes induced by IR**

(A) Protein band described at *Figure* 2A was excised, digested with trypsin and subjected to MALDI-TOF analysis (Microflex LRF 20). Spectra were collected and calibrated using Trypsin auto-digestion peaks (m/z 842.5099, 2211.1046). (B-D) The histological changes of diameter were assessed in control, GBM xenograft, GBM xenograft and irradiated mice. For measurement of muscle size, 8 mice per group were sacrificed and gastrocnemius were isolated in both legs. (B) The mouse skeletal muscles were photographed. (C) The gastrocnemius diameter was measured. (D) The weight of the gastrocnemius was measured. (E-J) Normal mice induced muscle atrophy via irradiation. (E) 8 normal mice were irradiated (total 10 Gy, 2 Gy for 5 days) and body weight were analyzed after 3 weeks. (F) Gastrocnemius were stained with hematoxylin and eosin (H&E) to demonstrate the extent of the muscle atrophy.

Photomicrographs were taken at 20x magnification. Scale bars, 100 μm. (G) Myofiber cross-sectional area size in gastrocnemius was measured by ImageJ. (H) The gastrocnemius diameter was measured. (I) The weight of the gastrocnemius was measured. Statistical analysis was performed with one-way ANOVA plus a Tukey's multiple comparisons test. ** $p < 0.001$, ****$p < 0.0001$.

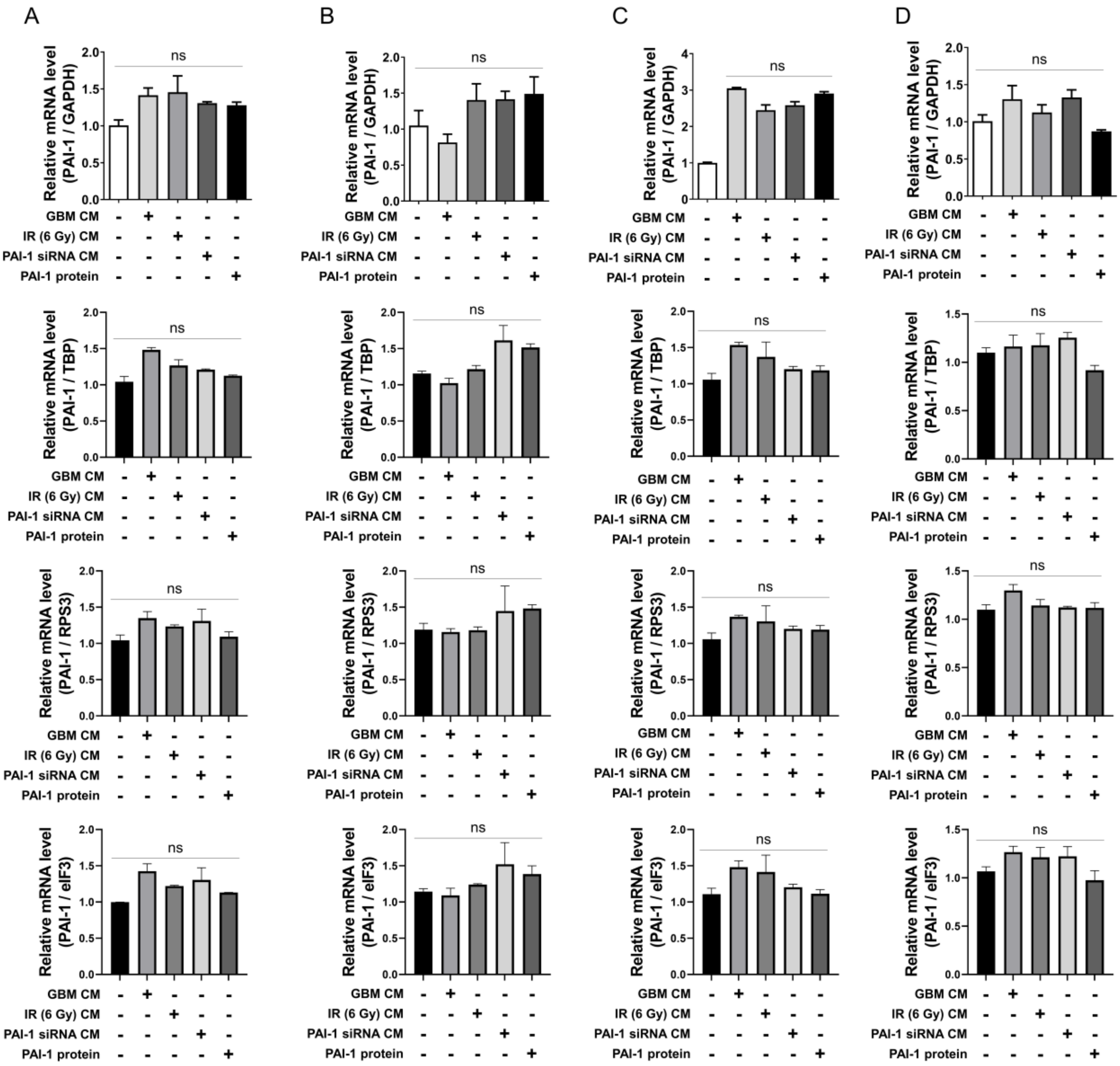


**Figure S2. IR induces upregulation of PAI-1 secreted from GBM cells induce cachexia phenotype in C2C12 myoblasts and differentiated myotubes**

(A-B) The mRNA expressions change of PAI-1 in C2C12 myoblast cell treated with U87MG CM in short term (incubated for 3 days with exosome), long term (incubated for 10 days with exosome) was assessed by qRT-PCR normalized with GAPDH (first panel), TBP (second panel), RPS3 (third panel), eIF3 (fourth panel). (C-D) The mRNA expressions change of PAI-1 in C2C12 differentiated myotube cell treated with U87MG CM in short term (incubated for 3 days with exosome), long term (incubated for 10 days with exosome) was assessed by qRT-

PCR normalized with GAPDH (first panel), TBP (second panel), RPS3 (third panel), eIF3 (fourth panel).

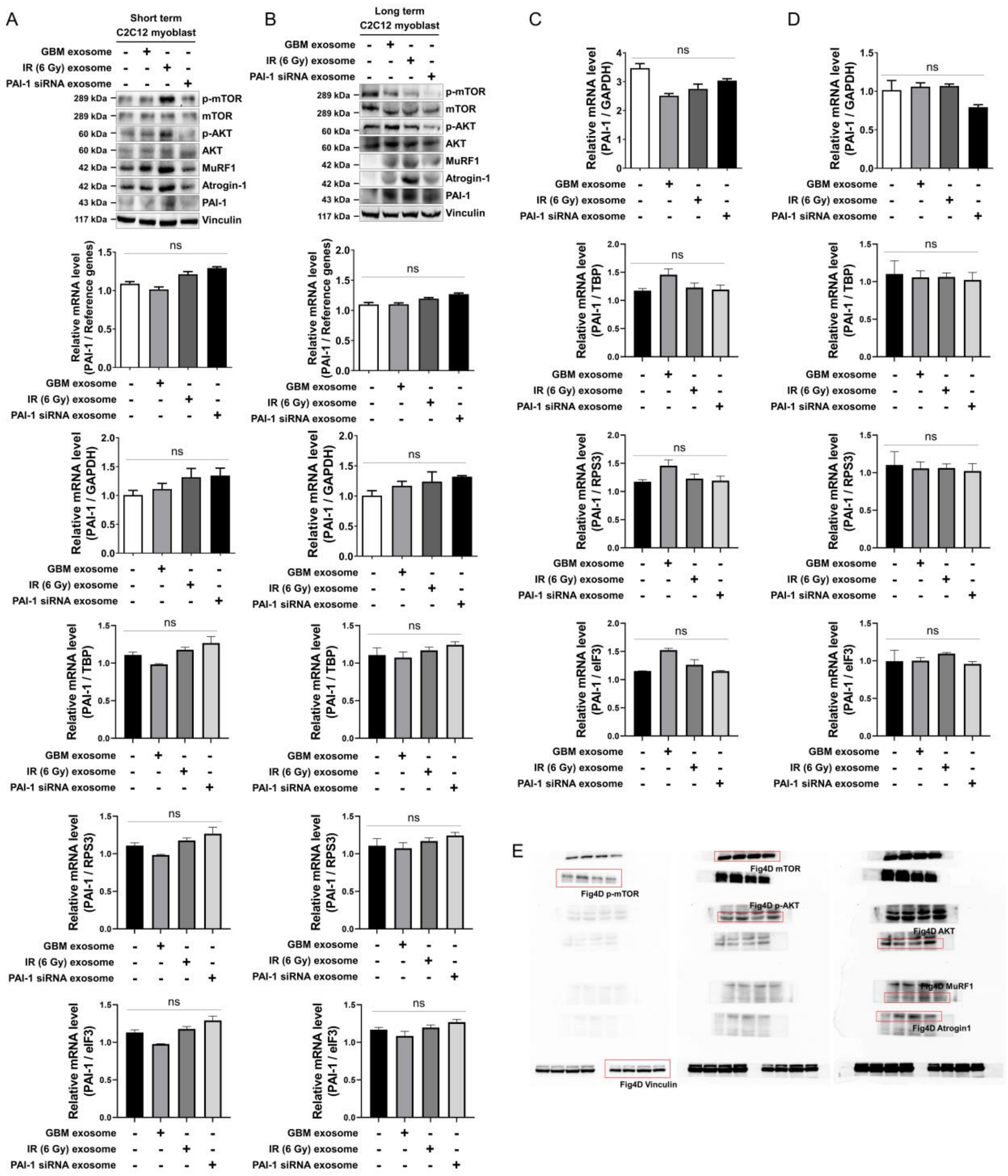

**Figure S3. IR induces upregulation of PAI-1 secreted from GBM cells induce cachexia phenotype in myoblasts and differentiated myotubes**

(A-B) The protein expression of muscle protein synthesis (p-mTOR, p-AKT), muscle protein degradation (MuRF1, Atrogin-1) related proteins and PAI-1 protein in C2C12 myoblast cell

treated with U87MG exosome in short term (incubated for 3 days with exosome), long term (incubated for 10 days with exosome) were assessed by western blot (first panel), mRNA expressions change of PAI-1 in C2C12 myoblast cell treated with U87MG exosome in short term (incubated for 3 days with exosome), long term (incubated for 10 days with exosome) were assessed by qRT-PCR (second panel). qRT-PCR was normalized with GAPDH (third panel), TBP (fourth panel), RPS3 (fifth panel), eIF3 (sixth panel). (C-D) PAI-1 mRNA expressions change in C2C12 differentiated myotube cell treated with U87MG exosome in short term (incubated for 3 days with exosome), long term (incubated for 10 days with exosome) were assessed by qRT-PCR normalized with GAPDH (first panel), TBP (second panel), RPS3 (third panel), eIF3 (fourth panel). (E) Unprocessed original image of western blots for Figure 4D data.

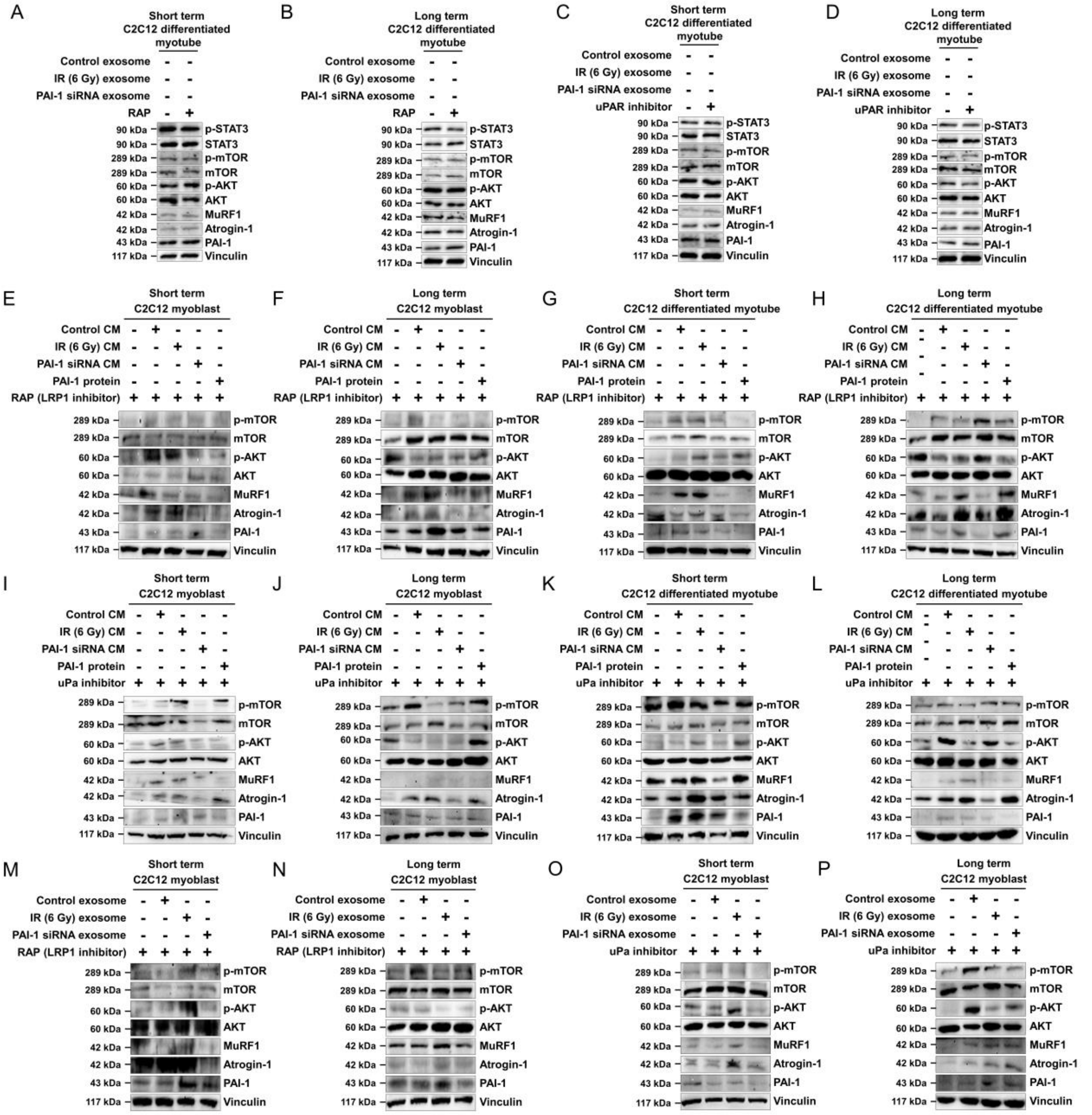


**Figure S4. Secreted PAI-1 and exosomal PAI-1 was directly penetrated via non-receptor-mediated endocytosis in myoblasts and differentiated myotubes**

(A-D) The protein expression of muscle protein synthesis (p-mTOR, p-AKT), muscle protein degradation (MuRF1, Atrogin-1) related proteins and PAI-1 protein in C2C12 differentiated myotube treated with LRP1 inhibitor (RAP) or uPAR inhibitor in short term, long term were assessed by western blot. (E-H) The protein expression of muscle protein synthesis (p-mTOR,

p-AKT), muscle protein degradation (MuRF1, Atrogin-1) related proteins and PAI-1 protein in C2C12 myoblast cell, C2C12 differentiated myotube treated with U87MG CM and LRP1 inhibitor (RAP) in short term, long term were assessed by western blot. (I-L) The protein expression of muscle protein synthesis (p-mTOR, p-AKT), muscle protein degradation (MuRF1, Atrogin-1) related proteins and PAI-1 protein in C2C12 myoblast cell, C2C12 differentiated myotube treated with U87MG CM and uPa inhibitor in short term, long term were assessed by western blot. (M-N) The protein expression of muscle protein synthesis (p-mTOR, p-AKT), muscle protein degradation (MuRF1, Atrogin-1) related proteins and PAI-1 protein in C2C12 myoblast cell treated with U87MG exosome and LRP1 inhibitor (RAP) in short term, long term were assessed by western blot. (O-P) The protein expression of muscle protein synthesis (p-mTOR, p-AKT), muscle protein degradation (MuRF1, Atrogin-1) related proteins and PAI-1 protein in C2C12 myoblast cell treated with U87MG exosome and uPa inhibitor in short term, long term were assessed by western blot.

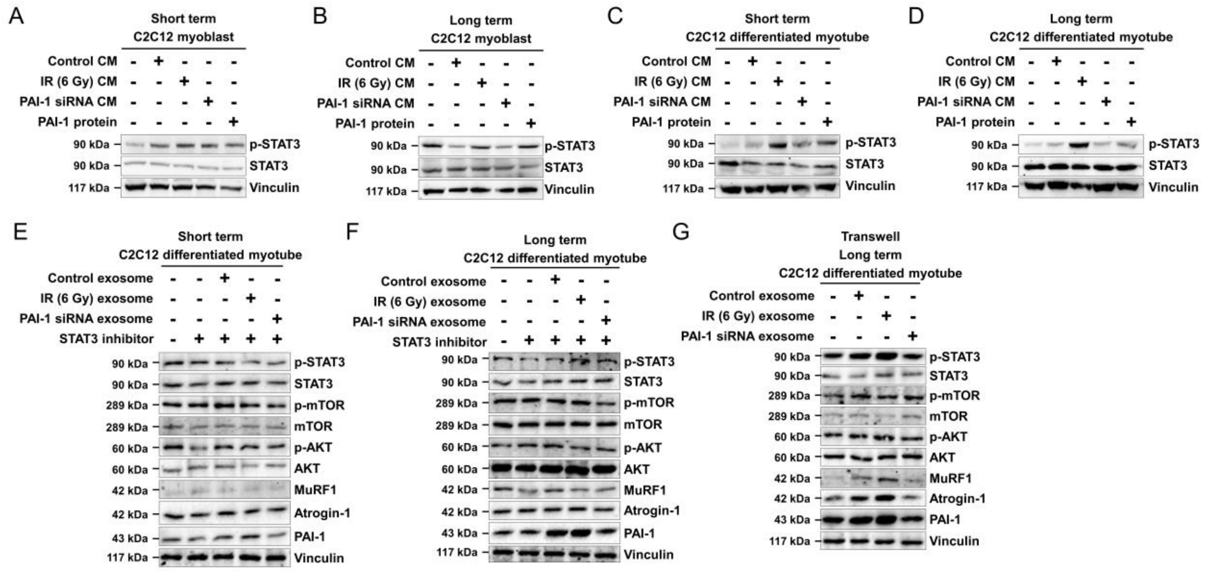


**Figure S5. Secreted PAI-1 and exosomal PAI-1 activated intracellular STAT3 in myoblasts and differentiated myotubes**

(A-D) The protein expression of phosphorylated STAT3 was assessed by western blot in C2C12 myoblast cell, C2C12 differentiated myotube cell treated with U87MG CM in short term, long term. (E-F) The protein expression of cachectic phenotypes was assessed by western blot in C2C12 differentiated myotube treated with U87MG exosome and STAT3 inhibitor in short term, long term. (G) The protein expression of cachectic phenotypes was assessed by western blot in C2C12 differentiated myotube treated with U87MG exosome in long term on transwell plate.

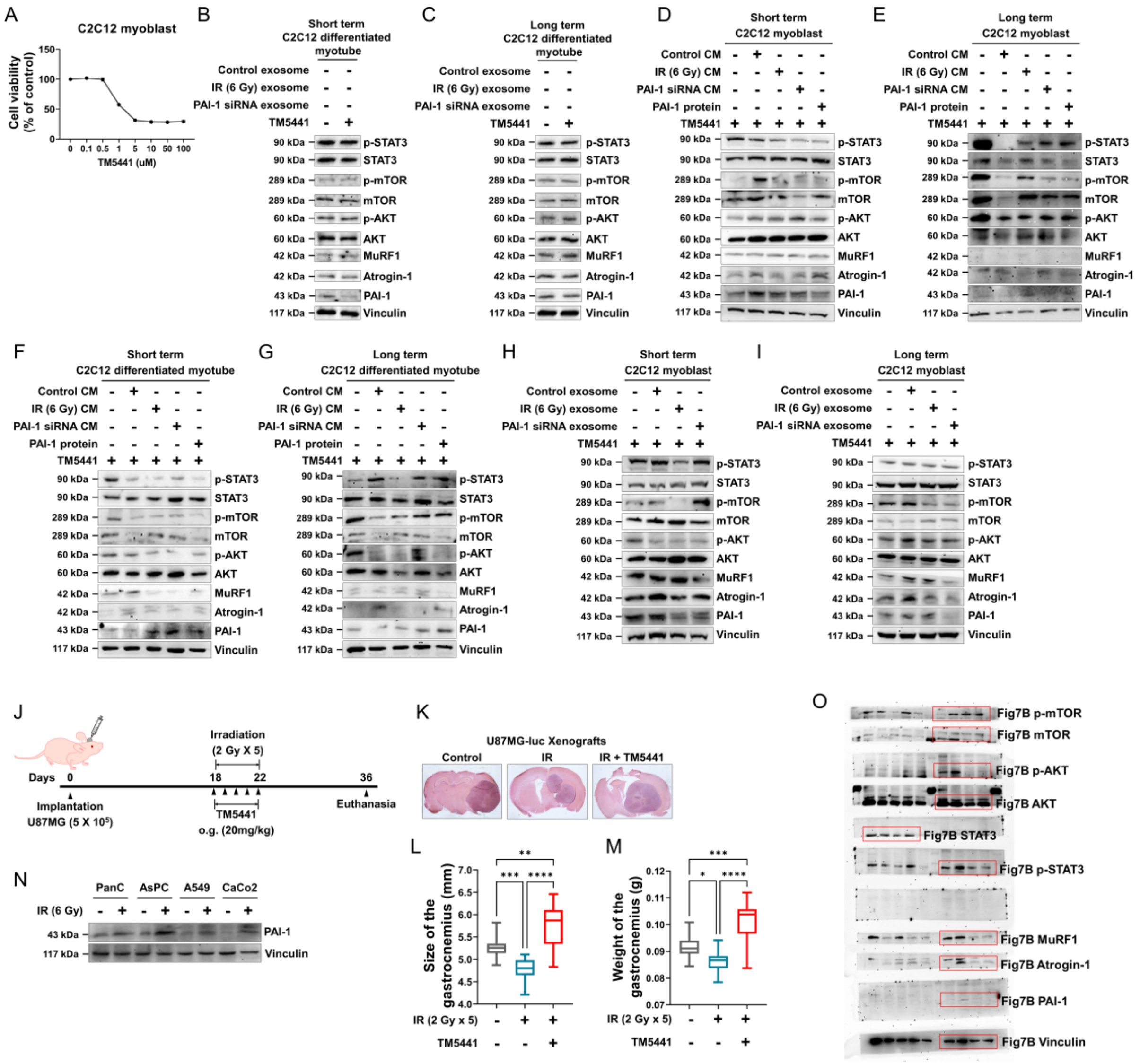

**Figure S6. Pharmacological inhibition of PAI-1 rescued IR-induced GBM cachexia**

(A) The effect of different concentrations of TM5441 on C2C12 myoblast cell viability. TM5441 did not reduce cell viability substantially at concentrations up to 0.5 μM. (B-C) The protein expression of cachectic phenotypes was assessed by western blot in C2C12 differentiated myotube treated with U87MG exosome and TM5441 in short term, long term. (D-G) The protein expression of muscle protein synthesis (p-mTOR, p-AKT), muscle protein degradation (MuRF1, Atrogin-1) related proteins and PAI-1 protein in C2C12 myoblast cell, C2C12 differentiated myotube treated with U87MG CM and TM5441, PAI-1 inhibitor in short

term, long term were assessed by western blot. (H-I) The protein expression of muscle protein synthesis (p-mTOR, p-AKT), muscle protein degradation (MuRF1, Atrogin-1) related proteins and PAI-1 protein in C2C12 myoblast cell treated with U87MG exosome and TM5441, PAI-1 inhibitor in short term, long term were assessed by western blot. (J) A scheme diagram of control, IR (2 Gy X 5) and IR with TM5441 (20mg/kg, oral) treatment in mice bearing U87MG-luciferase xenografts (n=12 mice per group). (K) Hematoxylin and eosin (H&E) staining of coronal sections from mice bearing U87MG-luciferase xenografts control, treated with IR or IR with TM5441. Scale bars, 250 μm. (L) Size of gastrocnemius muscle from mice bearing U87MG-luciferase xenografts control, treated with IR or IR with TM5441. (M) Weights of gastrocnemius muscle from mice bearing U87MG-luciferase xenografts control, treated with IR or IR with TM5441. (N) Protein levels of PAI-1 and Vinculin were analyzed by western blot at 24hr after IR (6 Gy) in PanC, AsPC, A549, CaCo2 cells. (O) Unprocessed original image of western blots for Figure 7B data. Statistical analysis was performed with one-way ANOVA plus a Tukey's multiple comparisons test. * $p < 0.5$, ** $p < 0.05$, ***$p < 0.001$, ****$p < 0.0001$, ns Not significant.

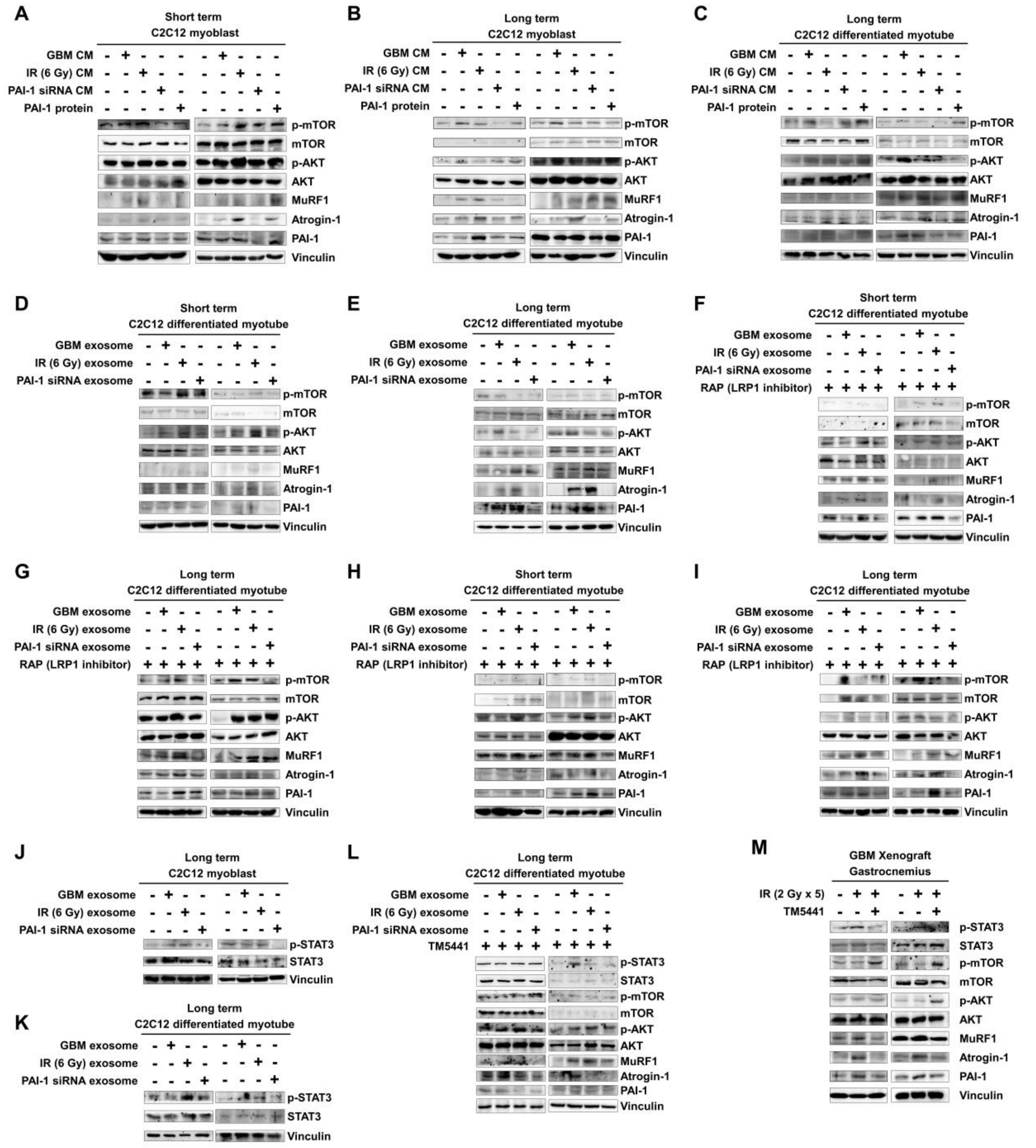


**Figure S7. Replicate data of western blot analysis**

(A) Replicate data of Figure 3A western blot analysis. (B) Replicate data of Figure 3B western blot analysis. (C) Replicate data of Figure 3D western blot analysis. (D) Replicate data of Figure 4C western blot analysis. (E) Replicate data of Figure 4D western blot analysis. (F) Replicate data of Figure 5A western blot analysis. (G) Replicate data of Figure 5B western blot

analysis. (H) Replicate data of Figure 5C western blot analysis. (I) Replicate data of Figure 5D western blot analysis. (J) Replicate data of Figure 6B western blot analysis. (K) Replicate data of Figure 6D western blot analysis. (L) Replicate data of Figure 7B western blot analysis. (M) Replicate data of Figure 7H western blot analysis.
